# Androgen Receptor‐Induced Lactoferrin Accelerates Prostate Tumorigenesis Through Modulating Ferroptosis

**DOI:** 10.1002/advs.202520109

**Published:** 2026-04-07

**Authors:** Can Liu, Qiu Peng, Xiaoyue Zhang, Zhengshuo Li, Yangge Wu, Yuqing Wen, Run Zheng, Shuanglong Wu, Chenfei Zhou, Zhengyu Hu, Lanjun Shu, Xinyi Ma, Qiuyang Li, Ya Ma, Chunlin Ou, Ming Zhao, Songqing Fan, Lingyu Wei, Jian Ma

**Affiliations:** ^1^ Hunan Key Laboratory of Cancer Metabolism Hunan Cancer Hospital/ The Affiliated Cancer Hospital of Xiangya School of Medicine Central South University Changsha Hunan China; ^2^ Cancer Research Institute Xiangya School of Basic Medical Science Central South University Changsha Hunan China; ^3^ Ningbo Clinical Pathology Diagnosis Center Ningbo Zhejiang China; ^4^ Key Laboratory of Carcinogenesis and Cancer Invasion of the Chinese Ministry of Education NHC Key Laboratory of Carcinogenesis Hunan Key Laboratory of Nonresolving Inflammation and Cancer Furong Laboratory Changsha Hunan China; ^5^ Shanxi Provincial Center For Upper Gastrointestinal Cancer Research and Clinical Translation Laboratory of Clinical Research Center Heping Hospital Affiliated to Changzhi Medical College Changzhi Shanxi China; ^6^ Department of Pathology Xiangya Hospital Central South University Changsha Hunan China; ^7^ Department of Pathology Second Xiangya Hospital Central South University Changsha Hunan China

**Keywords:** androgen receptor, ferroptosis, lactoferrin, prostate cancer, TRAMP mouse model

## Abstract

Lactoferrin (LF), an innate immunity molecule, showed a strikingly high expression level in the human prostate compared to other tissues and organs, indicating a significant role in prostate physiology. Despite the tumor‐suppressive role of lactoferrin established in other malignancies, we reveal its paradoxical oncogenic function in prostate cancer through an androgen receptor (AR)‐LF‐ferroptosis axis. Utilizing *Lf ^−/−^* TRAMP genetic mouse models, proteomics, TCGA‐PARD data, and single‐cell RNA‐seq, we demonstrate that AR directly binds the *LF* promoter, driving *LF* expression, which in turn upregulates ferritin (FTH1/FTL) expression and suppresses p53‐ALOX12‐mediated ferroptosis in prostate cancer. Crucially, *Lf* deficiency delayed tumor progression and intensified ferroptotic stress in the TRAMP mice, while iron supplementation accelerated carcinogenesis—effects rescued by *Lf* knockout. Mechanistically, lactoferrin shields prostate cancer cells from iron‐induced ferroptosis by maintaining iron‐redox homeostasis. Preclinical targeting of this axis suggested a potential therapeutic strategy, as suppressed tumor growth in prostate cancer xenograft was observed following *LF* knockdown coupled with ferroptosis induction (via IKE) and androgen receptor inhibition (via enzalutamide). This work defines lactoferrin as: (i) an AR‐regulated ferroptosis suppressor, (ii) a regulator of prostate cancer's “iron addiction,” and (iii) a candidate target for therapeutic exploitation of iron‐metabolic vulnerability.

## Graphical abstract

This study demonstrates that transcription factor androgen receptor (AR) directly binds the *LF* promoter, driving lactoferrin overexpression to promote ferritin (FTH1/FTL) upregulation and inhibit p53‐ALOX12‐mediated ferroptosis in prostate cancer. Lactoferrin could be a new potential therapeutic target in prostate cancer.

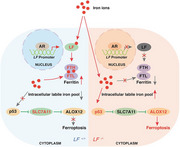

## 1 Introduction

Androgen receptor (AR) plays a crucial role in prostate cancer development and progression, which has positioned AR‐axis targeting as the cornerstone of clinical management for over seven decades. AR, a ligand‐dependent transcription factor belonging to the nuclear receptor superfamily, dimerizes upon binding to dihydrotestosterone (DHT) and translocates into the nucleus to regulate target gene expression [1]. Since Huggins' landmark discovery of androgen deprivation therapy (ADT) in 1941, therapeutic strategies have evolved through generations of AR‐targeting agents—from first‐generation anti‐androgens (e.g., bicalutamide) to novel androgen synthesis inhibitors (abiraterone) and next‐generation AR antagonists (enzalutamide/apalutamide) [2, 3, 4]. As the oncogenic driver in lethal prostate cancers, unraveling the molecular mechanisms of AR signaling during prostate cancer initiation and progression is imperative to identify novel therapeutic strategies with significant clinical implications [5].

Located at chromosome 3p21.31, the human *Lactoferrin* (*LF*) gene, a member of the transferrin family, is essential for the maintenance of iron metabolism, cellular signaling, and redox homeostasis [6]. Genetic linkage analysis of 18 multigenerational nasopharyngeal carcinoma (NPC) pedigrees from Hunan Province, China, our laboratory identified an NPC susceptibility locus on chromosome 3p21 [7], and *LF* was the top‐ranked candidate down‐regulated gene within this region [7, 8, 9, 10]. Subsequent functional analysis revealed that lactoferrin antagonizes Epstein‐Barr virus (EBV)‐induced inflammation by inhibiting Toll‐like receptor 2 and 9 (TLR2/TLR9) activation, implicating its potential role in NPC prevention [11, 12]. In previous investigations, we constructed the *Lf* gene knockout mouse and demonstrated that lactoferrin suppresses tumor progression in the AOM/DSS‐induced colitis‐associated colorectal cancer model [13], as well as in the B16 melanoma metastasis model by neutralizing myeloid‐derived suppressor cell (MDSC) functionality [14]. Lactoferrin is a secretory glycoprotein and innate immune factor, and exhibits high expression levels in glandular tissues and is found at high concentrations in exocrine secretions, such as milk and semen [15]. In the prostate tissue, lactoferrin is predominantly produced by epithelial cells within the central zone, periurethral glandular epithelium, and prostatic urethral epithelium. Given the observed high abundance of lactoferrin expression in the prostate tissues, we wanted to find out what role lactoferrin plays in the development of prostate cancer.

Ferroptosis represents a mechanistically distinct iron‐dependent cell death, fundamentally divergent from canonical pathways including apoptosis, necrosis, and autophagy. Its molecular hallmarks comprise:(i) expansion of redox‐active labile Fe^2^^+^ pools; (ii) pathological accumulation of phospholipid peroxidation products (e.g., MDA/4‐HNE); (iii) compromised antioxidant defenses—notably glutathione (GSH) depletion and GPX4 inactivation. Tumor cells exhibit a dualistic dependence on iron: while sustained proliferation demands abundant iron as an essential cofactor—manifesting as profound iron avidity—concomitantly elevated labile Fe^2^^+^ pools generate oxidation‐driven cytotoxicity that licenses ferroptotic death. Lactoferrin exhibits a high‐affinity iron‐binding capacity that exceeds transferrin (TF) by 250–300‐fold, enabling potent Fenton reaction inhibition that underlies multifunctional bioactivities, including immunomodulation and antioxidant defense [16].

In this study, to delineate the functional role of lactoferrin in prostate carcinogenesis, we crossed *Lf* knockout mouse (*Lf ^−/−^* established by our laboratory) [13, 14]with the transgenic adenocarcinoma of the mouse prostate model (TRAMP) [17, 18]. Contrary to expectations that lactoferrin acts as a tumor suppressor, *Lf ^−/−^* TRAMP mice exhibited significant tumor developmental delay through induction of ferroptosis. Our study demonstrates that transcription factor AR‐regulated LF promotes ferritin (FTH1/FTL) upregulation and confers resistance to ferroptosis in prostate cancer cells by inhibiting the p53‐ALOX12 signaling pathway.

## 2 Results

### 2.1 Lactoferrin Deficiency Delays Prostate Tumorigenesis in TRAMP Mice

Lactoferrin, a secreted protein, is widely distributed across tissues and organs via glandular fluids, influencing physiological and pathological processes through intercellular signaling, immunomodulation, and cation transport [19]. Interrogation of *The Human Protein Atlas* database revealed strikingly high lactoferrin expression levels in the human prostate (Figure S1A). Retrospective analysis of the formalin‐fixed paraffin‐embedded biopsies (FFPE_Bx) cohort revealed that elevated lactoferrin expressions were significantly associated with shorter disease‐free survival (Figure S1B), suggesting a link between high lactoferrin levels and poor prognosis of prostate cancer patients [20]. Analysis of clinical cohorts linked high lactoferrin expression to prostate cancer progression. In the MSKCC_2010 cohort [21], lactoferrin expressions were significantly higher in patients with elevated Gleason scores (Figure S1C). Moreover, in the PRAD_FHCRC cohort [22], lactoferrin expressions were markedly increased in bone metastasis samples relative to primary tumors (Figure S1D).

**FIGURE 1:**
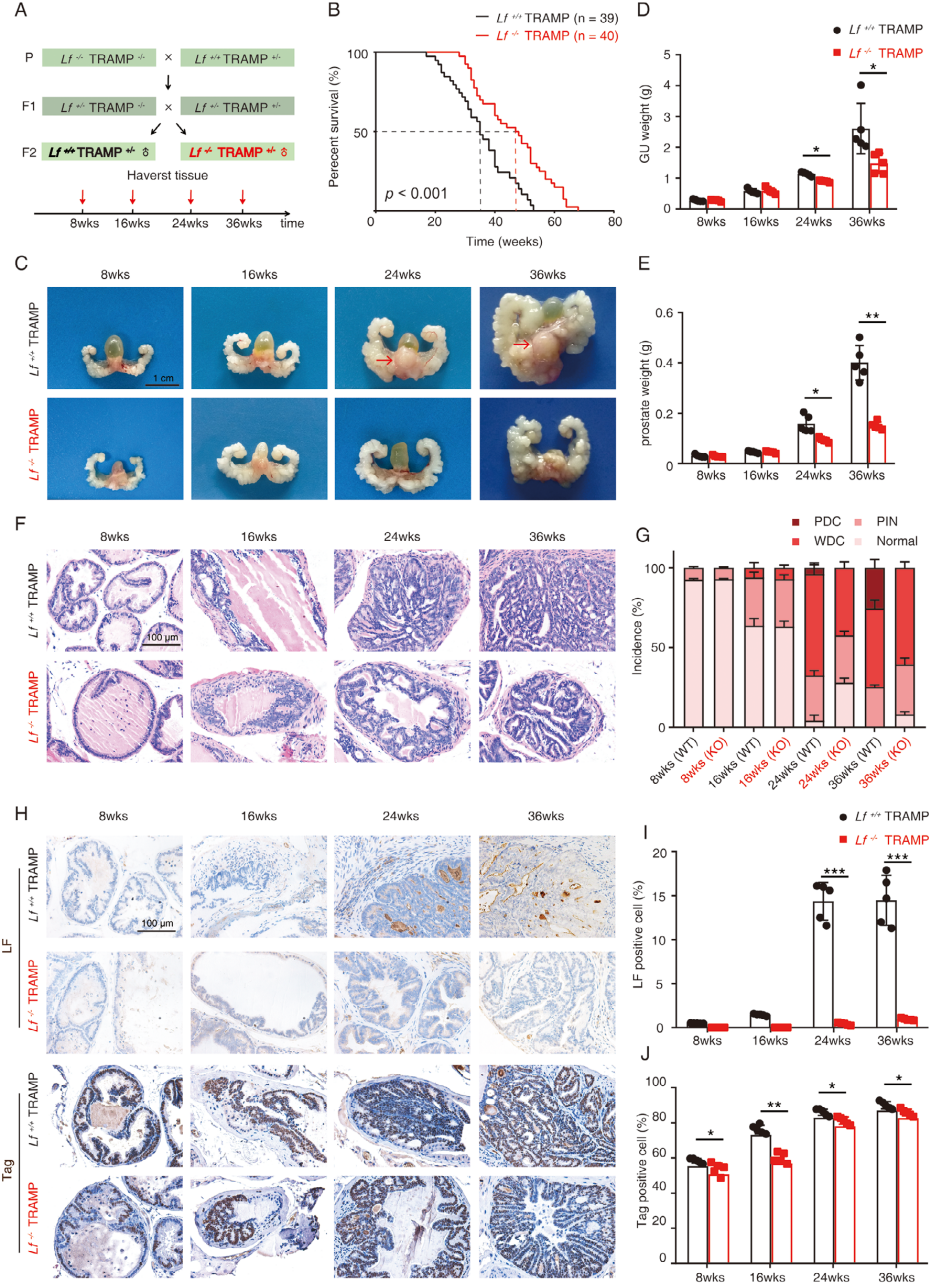
Lactoferrin deficiency delays tumorigenesis in TRAMP mouse prostate tissues. (A) Breeding scheme and tissue collection timeline for *Lf ^‐/−^* TRAMP and *Lf ^+/+^* TRAMP male mice. (B) Kaplan‐Meier survival curves and median survival comparison (*Lf ^‐/−^* TRAMP vs. *Lf ^+/+^* TRAMP). (C) Gross morphology of genitourinary (GU) and prostate tissues at 8, 16, 24, and 36 weeks. Red arrows indicate tumor foci. (D) Quantification of GU weights. (E) Quantification of prostate tissue weights. Data represent mean ± SD. (F) Histopathological evaluation of prostate tissues (H&E) at indicated timepoints. (G) Pathological grading statistics: PDC (poorly differentiated adenocarcinoma), WDC (well‐differentiated adenocarcinoma), PIN (prostatic intraepithelial neoplasia), WT: *Lf ^+/+^* TRAMP, KO: *Lf ^‐/−^* TRAMP. (H) Immunohistochemical staining of lactoferrin (LF) and T‐antigen (Tag) in prostate tissues. (I) and (J) Quantification of LF‐positive and Tag‐positive cells in prostate tissues. *n* = 5, each group. *p* < 0.05 “*,” *p* < 0.01 “**,” *p* < 0.001 “***” (one‐way ANOVA).

To delineate the in vivo functional contribution of lactoferrin to prostate carcinogenesis, *Lf* ^−/−^mice (previously established in our laboratory) were crossed with the well‐established transgenic adenocarcinoma of the mouse prostate TRAMP^+/−^ transgenic mice. This breeding protocol generated male experimental *Lf* ^−/−^ TRAMP^+/−^ mice (lactoferrin knockout, abbreviated as *Lf* ^−/−^ TRAMP) and their male *Lf* ^+/+^ TRAMP^+/−^ littermates (wild‐type lactoferrin, abbreviated as *Lf* ^+/+^ TRAMP) as controls (breeding scheme: Figure 1A; Genotype identification: Figure S2A). Animals were sacrificed at defined timepoints (8, 16, 24, and 36 weeks), corresponding to progressive tumor stages—assessment at each interval, including survival analysis, organ morphology, and histopathology. Survival analysis demonstrated significantly prolonged overall survival in *Lf* ^−/−^ TRAMP mice (*n *= 40) vs. *Lf* ^+/+^ TRAMP littermates (*n *= 39), with increases in both maximum (68 vs. 53 weeks) and median survival (47.5 vs. 37.5 weeks) (Figure 1B). At advanced‐stage (24 or 36 weeks), genitourinary (GU) morphology revealed that *Lf* ^+/+^ TRAMP mice developed macroscopic, smooth‐bordered prostatic tumors (Figure 1C, red arrows), while *Lf* ^−/−^ TRAMP mice exhibited significantly reduced genitourinary mass and prostate weight (Figure 1D,E). Though well‐differentiated adenocarcinoma (WDC) appeared in all cohorts, *Lf* ^+/+^ TRAMP prostate glands demonstrated more architectural disintegration than *Lf* ^−/−^ TRAMP at 24 weeks; besides, loss of glandular organization and progressed to poorly differentiated carcinoma (PDC) were appeared in *Lf* ^+/+^ TRAMP prostate glands, while *Lf* ^−/−^ TRAMP prostates remained at WDC stage (Figure 1F,G and Figure S2B,C). IHC analysis identified stage‐dependent lactoferrin upregulation in *Lf* ^+/+^ TRAMP prostates (24/36 weeks) concomitant with T‐antigen reduction in *Lf* ^−/−^ TRAMP (Figure 1H–J).

Comparative assessment of metastatic burden in *Lf* ^−/−^ TRAMP vs. *Lf* ^+/+^ TRAMP mice revealed macroscopic metastatic lesions in 36‐week *Lf* ^+/+^ TRAMP lungs (Figure S2D–F, 60% incidence) and kidneys (Figure S2H–J, 40% incidence), with complete absence of metastases in *Lf* ^−/−^ TRAMP counterparts. T‐antigen immunohistochemistry confirmed the prostatic origin of metastatic cells in *Lf* ^+/+^ TRAMP tissues (Figure S2G,K). Molecular analysis of 36‐week mouse prostate specimens demonstrated significant downregulation of proliferation marker Ki67, epithelial‐mesenchymal transition (EMT) regulator Snail, and Androgen receptor AR, indicating lactoferrin deficiency suppresses proliferative capacity, metastatic potential, and androgen signaling (Figure S2L–O). These observations revealed that lactoferrin deficiency delays prostate tumorigenesis in TRAMP mice.

### 2.2 Key Signaling Pathways Altered by Lactoferrin Expression in Prostate Cancer

**FIGURE 2:**
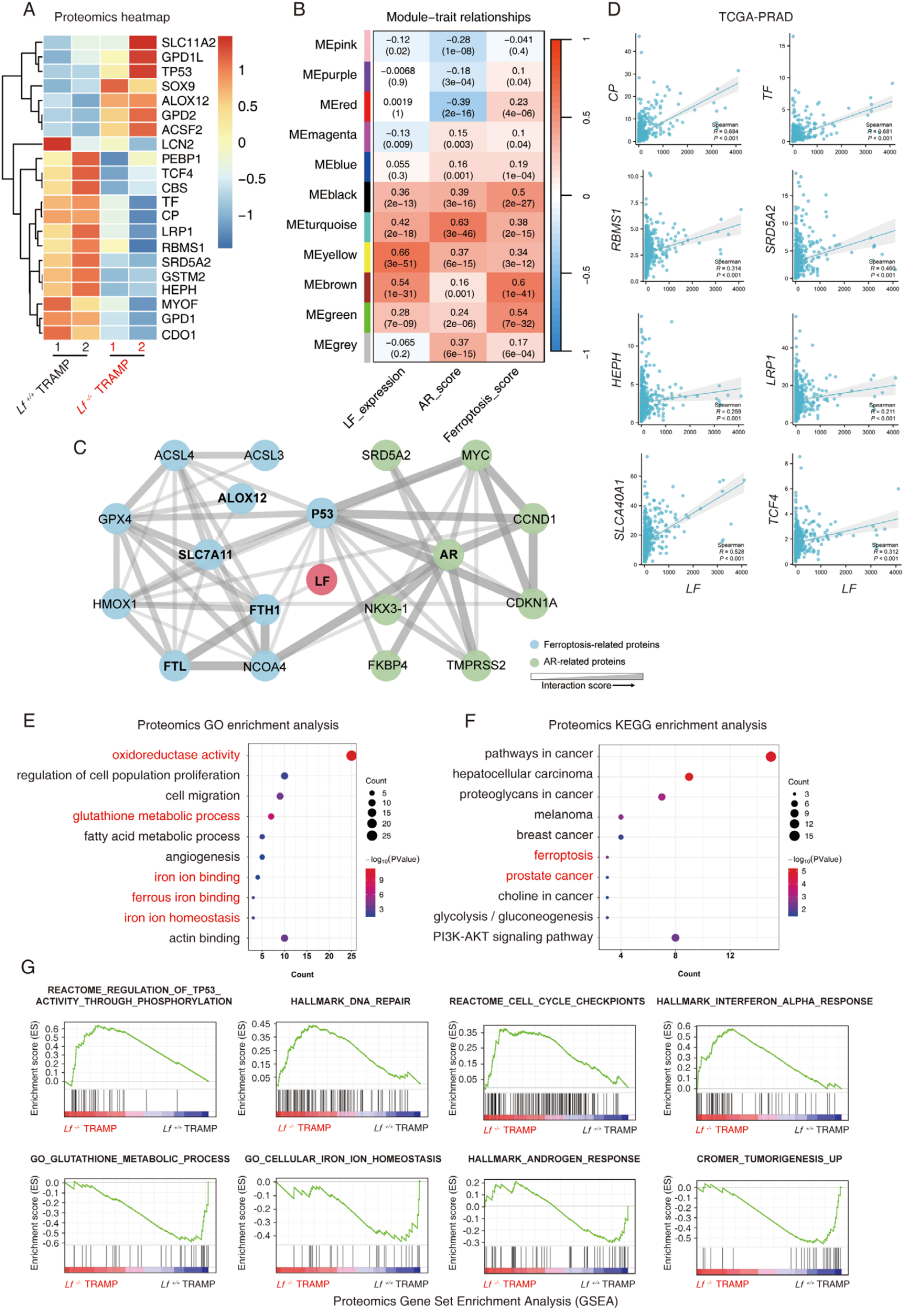
Identification of critical signaling pathways modulated by lactoferrin deficiency. (A) Heatmap of differentially expressed proteins (DEPs) in prostate tissues (*n* = 2) between *Lf ^+/+^* TRAMP and *Lf ^‐/−^* TRAMP mice at 36 weeks. (B) Heatmap of module‐trait relationships among “LF_expression,” “AR_score,” and “Ferroptosis_score” based on TCGA‐PRAD dataset. (C) Protein‐Protein Interaction (PPI) Networks among “LF,” “AR‐related proteins,” and “Ferroptosis‐related proteins.” (D) Correlation analysis between *LF* and key molecular features in the TCGA‐PRAD: iron metabolism genes (*CP*, *TF*, *HEPH*, *SLC40A2*), ferroptosis regulators (*RBMS1*, *TCF4*), the lactoferrin receptor (*LRP1*), and androgen‐activated signaling marker (*SRD5A2*). (E) GO functional enrichment of DEPs upregulated in *Lf ^+/+^* TRAMP group. (F) KEGG pathway enrichment of DEPs upregulated in *Lf ^+/+^* TRAMP group. (G) GSEA analysis of proteomic profiles: *Lf ^‐/−^* TRAMP vs. *Lf ^+/+^* TRAMP.

We were surprised that lactoferrin plays a role as a tumor promoter in the mouse prostate cancer model. To elucidate the molecular mechanisms underlying lactoferrin's role in prostate tumorigenesis, we conducted unbiased TMT‐based quantitative proteomics on 36‐week prostate tissues from *Lf* ^−/−^ (KO) TRAMP vs. *Lf* ^+/+^ (WT) TRAMP mice. Differential expression analysis (*p *< 0.05, FC >1.2) identified 266 proteins significantly upregulated in the *Lf* ^−/−^ TRAMP group and 282 proteins upregulated in the *Lf* ^+/+^ TRAMP group (Figure S3A). *Lf* ^−/−^‐enriched proteins demonstrated significant overrepresentation of ferroptosis drivers: TP53 [23], ALOX12 [23], and ACSF2 [24], *Lf* ^+/+^‐enriched proteins demonstrated significant overrepresentation of ferroptosis suppressors: CP [25], RBMS1 [26], and GPD1 [27] (Figure 2A, and Figure S3B). Parallel analysis of TCGA‐PRAD data stratified by *LF* expression (top vs. bottom 25%, *p *< 0.05, FC >1.2) revealed 1910 genes upregulated in the *LF* high cohort and 1,277 genes upregulated in the *LF* low cohort (Figure S3C). Weighted Gene Co‐expression Network Analysis (WGCNA) demonstrated module‐specific and significant correlations linking LF expressions, AR pathway activities, and ferroptosis features (Figure 2B,C, and Figure S3D–F). Correlation analysis demonstrated significant positive associations between *LF* expression and iron metabolism genes *CP, TF, HEPH*, and *SLC40A1/FPN*; lactoferrin receptor *LRP1*; ferroptosis suppressors *RBMS1, TCF4*; and androgen activator *SRD5A2* (Figure 2D). Integrated GO/KEGG pathway analysis of “*Lf* ^+/+^‐upregulated proteins” or “*LF* high expression ‐upregulated genes” revealed significant enrichment in ferroptosis signaling, iron ion metabolism/transport, glutathione metabolism, and redox processes (Figure 2E,F, and Figure S3G,H). GSEA of TRAMP proteomic and TCGA‐PRAD transcriptomic profiles demonstrated p53 signaling, DNA repair, and cell cycle checkpoints were enriched in *Lf* ^−/−^ cohort; glutathione metabolism, iron ion homeostasis, and androgen response, tumorigenesis pathways were enriched in *Lf* ^+/+^ cohort (Figure 2G and Figure S3I).

### 2.3 Lactoferrin Deficiency Enhances Ferroptosis Vulnerability in TRAMP Prostate Tissue

**FIGURE 3:**
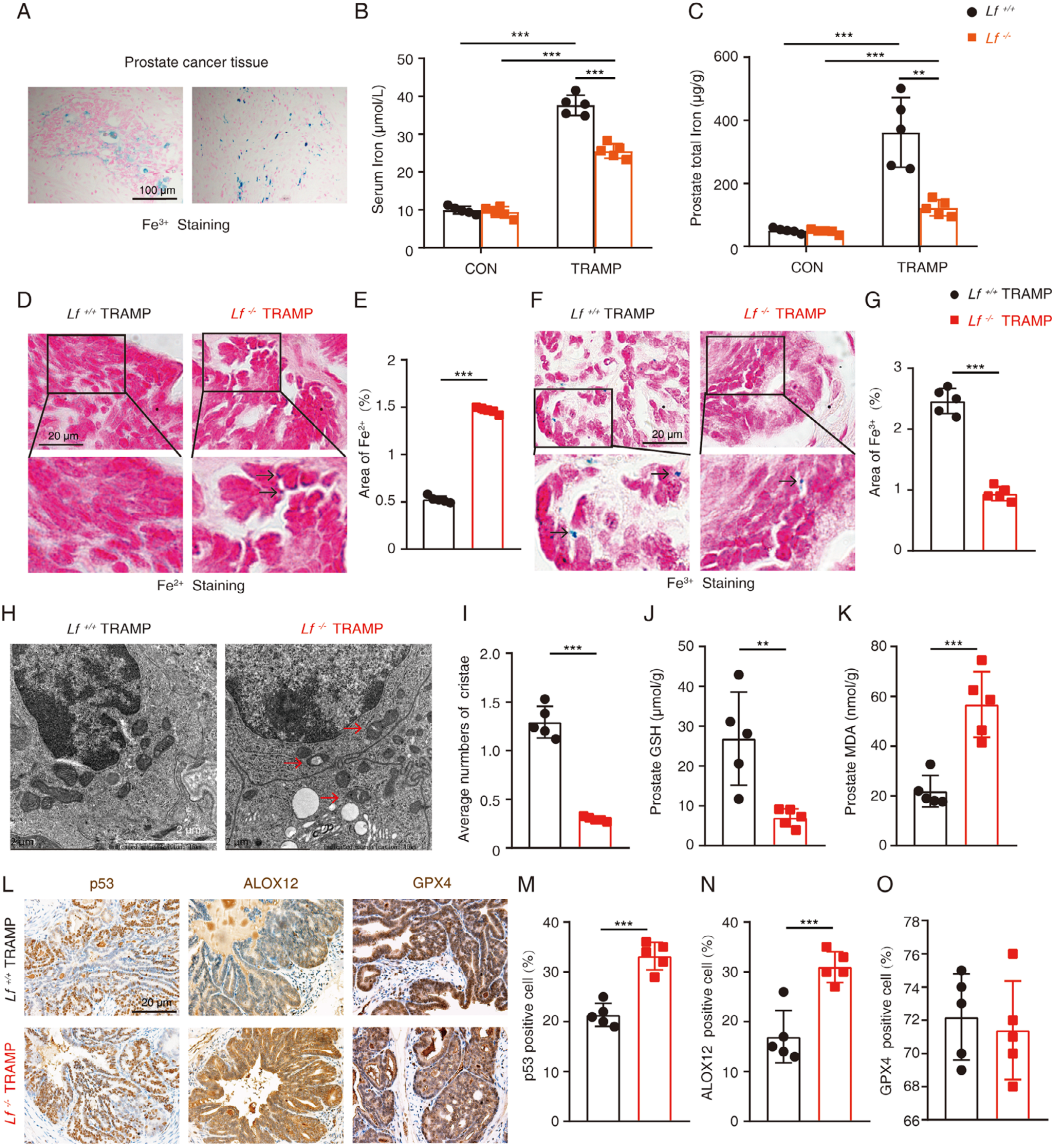
Lactoferrin deficiency enhances ferroptosis stress in TRAMP mouse prostate tissues. (A) Fe^3^^+^ staining (Prussian blue) in human prostate cancer tissues. (B) and (C) Iron ion levels in serum and prostate tissues of 36‐week‐old TRAMP mice and controls (CON). Both CON and TRAMP groups include *Lf ^+/+^* and *Lf ^‐/−^* mice. (D) Fe^2^^+^ staining (Turnbull's blue) in prostate tissues of *Lf ^+/+^* TRAMP and *Lf ^‐/−^* TRAMP mice at 36 weeks. Arrows indicate Fe^2^^+^‐positive areas. (E) Quantification of Fe^2^^+^‐positive area percentage. (F) Fe^3^^+^ staining in prostate tissues. Arrows indicate Fe^3^^+^‐positive areas. (G) Quantification of Fe^3^^+^‐positive area percentage. (H) Transmission electron microscopy images of mitochondrial morphology in mouse prostate tissues, and red arrows indicate shrunken mitochondria with reduced cristae. (I) Quantitative analysis of mitochondrial cristae number per mitochondrion. (J) Glutathione (GSH) content in prostate tissues. (K) Malondialdehyde (MDA) content in prostate tissues. (L) Immunohistochemical staining of p53, ALOX12, and GPX4 in prostate tissues. (M‐O), Quantitative analysis of p53‐, ALOX12‐, and GPX4‐positive cells. *n* = 5, *p* < 0.01 “**,” *p* < 0.001 “***” (one‐way ANOVA for multi‐group comparisons; Student's *t*‐test for two‐group comparisons).

Prussian blue staining revealed substantial ferric iron (Fe^3^^+^) deposition within mouse prostate carcinoma tissues (Figure 3A). Iron concentrations in serum and prostate tissues of TRAMP mice compared to non‐tumor controls were significantly elevated (TRAMP vs. CON), and *Lf* ^−/−^ TRAMP mice showed markedly reduced serum and prostate tissue iron levels relative to *Lf* ^+/+^ TRAMP littermates (Figure 3B,C). Histochemical analysis further revealed increased ferrous iron (Fe^2^^+^) accumulation (Figure 3D,E) alongside decreased Fe^3^^+^ accumulation (Figure 3F,G) in *Lf* ^−/−^ TRAMP (compared to *Lf* ^+/+^ TRAMP littermates) prostates, indicating an altered iron redox homeostasis by *Lf* deficiency. Transmission electron microscopy identified characteristic ferroptotic ultrastructural alterations in *Lf* ^−/−^ TRAMP prostates, including mitochondrial cristae dissolution (Figure 3H,I). Biochemical analysis confirmed the decreased glutathione (GSH) and elevated malondialdehyde (MDA) levels in *Lf* ^−/−^ TRAMP mice (Figure 3J,K). Immunohistochemistry demonstrated significant upregulation of pro‐ferroptotic mediators p53 and ALOX12 in *Lf* ^−/−^ TRAMP mice compared to *Lf* ^+/+^ TRAMP littermates (Figure 3L–N), while GPX4 (Figure 3L,O) expression remained unchanged. These findings suggest that prostate tumor tissue is more susceptible to ferroptosis in the context of lactoferrin deficiency.

### 2.4 Lactoferrin Localizes Predominantly to Epithelial Cells in Prostate Tissue

**FIGURE 4:**
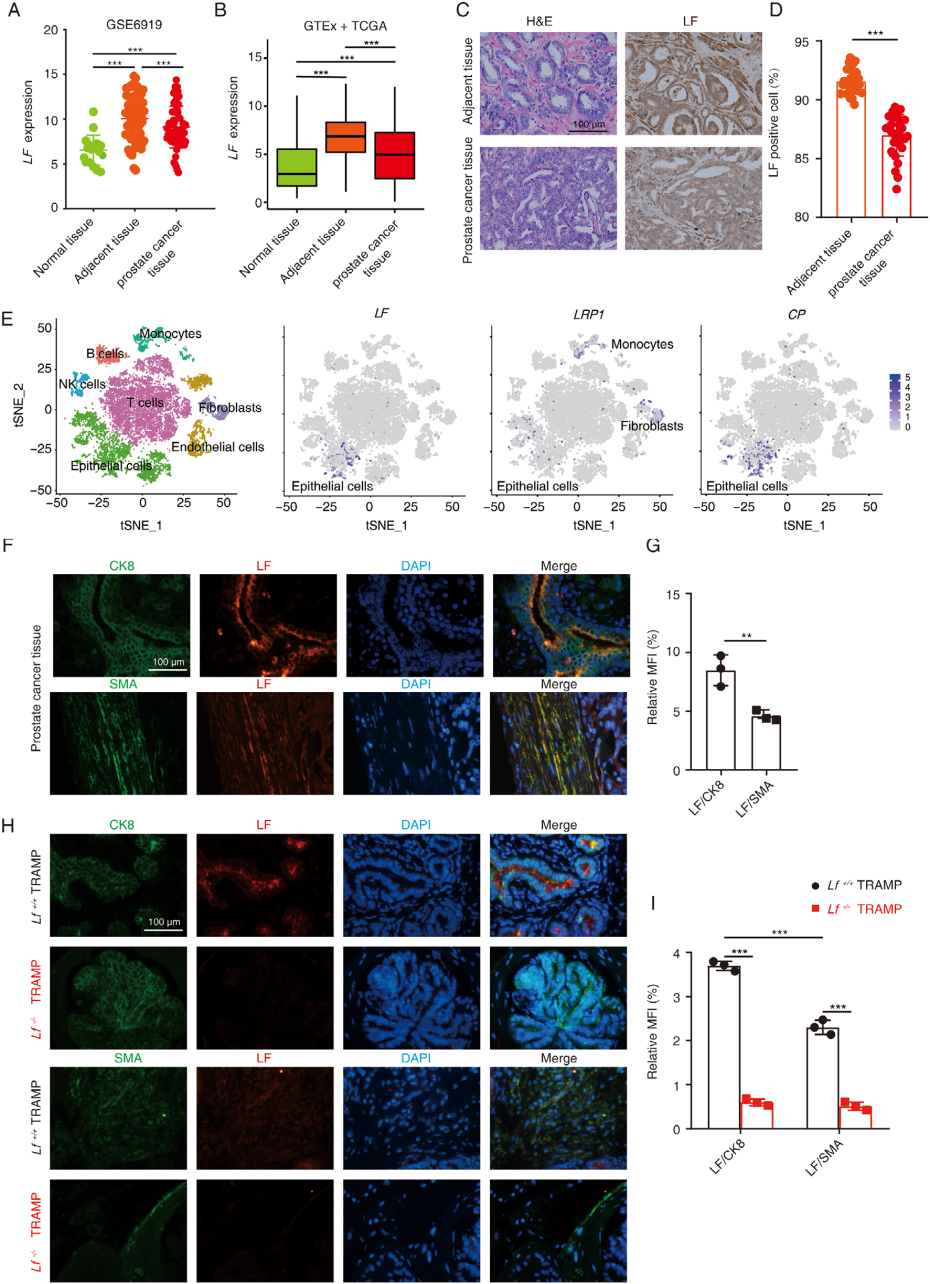
Spatial expression profiling of lactoferrin in prostate tumor microenvironment. (A) *LF* expression analysis in GSE6919: normal prostate (*n *= 18), adjacent tissues (*n *= 63), prostate cancer tissues (*n *= 65). (B) *LF* expression in GTEx‐TCGA integrated data: normal prostate (*n *= 100), adjacent tissues (*n *= 52), prostate cancer tissues (*n *= 499). (C) Immunohistochemical staining of LF in paired human tumor/adjacent tissues (*n *= 30). (D) Percentage of LF‐positive cells in tumor vs. adjacent tissues. (E) t‐SNE projection of human prostate cancer single‐cell clusters with *LF*, *LRP1*, and *CP* expression mapping. (F) Dual immunofluorescence of LF with CK8^+^ epithelial cells/SMA^+^ fibroblasts in human prostate cancer tissues (*n* = 3). (G) Quantification of LF Mean fluorescence intensity (MFI) in human epithelial vs. fibroblastic cells. (H) Dual immunofluorescence of LF with CK8^+^ epithelial cells/SMA^+^ fibroblasts in 36‐week TRAMP mouse prostate tissues (*n *= 3). (I) MFI analysis of LF in TRAMP epithelial/ fibroblastic cells. *p* < 0.01 “**,” *p* < 0.001 “***” (one‐way ANOVA for multi‐group comparisons; Student's *t*‐test for two‐group comparisons).

Our data revealed lactoferrin as an oncogenic facilitator in prostate tumorigenesis—contradicting its conventional tumor‐suppressor paradigm—through suppressing ferroptotic stress in TRAMP models. Transcriptomic analysis (GSE6919 and TCGA/GTEx) demonstrated consistent lactoferrin upregulation across tissue compartments, with gradient elevation in tumor‐adjacent tissues relative to carcinoma foci relative to normal prostate (Figure 4A,B). Clinically validated in 30 patient specimens, lactoferrin exhibited predominant localization in tumor‐adjacent epithelia, persisting in malignant microenvironments despite diminished secretory capacity in transformed cells (Figure 4C,D). Single‐cell resolution (GSE193337) revealed that lactoferrin predominantly localizes to epithelial cells; its receptor *LRP1* is expressed in epithelia, fibroblasts, and monocytes; and the lactoferrin‐binding protein *CP* localizes to epithelia (Figure 4E). This cellular mapping prompted our investigation toward lactoferrin‐mediated intercellular crosstalk between producer (epithelial) and receiver (epithelial/ fibroblast) populations in human and murine prostate cancers. Immunofluorescence confirmed spatial co‐localization of lactoferrin with CK8^+^ epithelia and SMA^+^ fibroblasts; lactoferrin is mainly located in epithelial cells in prostate cancer tissues (Figure 4F–I).

### 2.5 Androgen Receptor Directly Regulates *LF* Gene Expression

**FIGURE 5:**
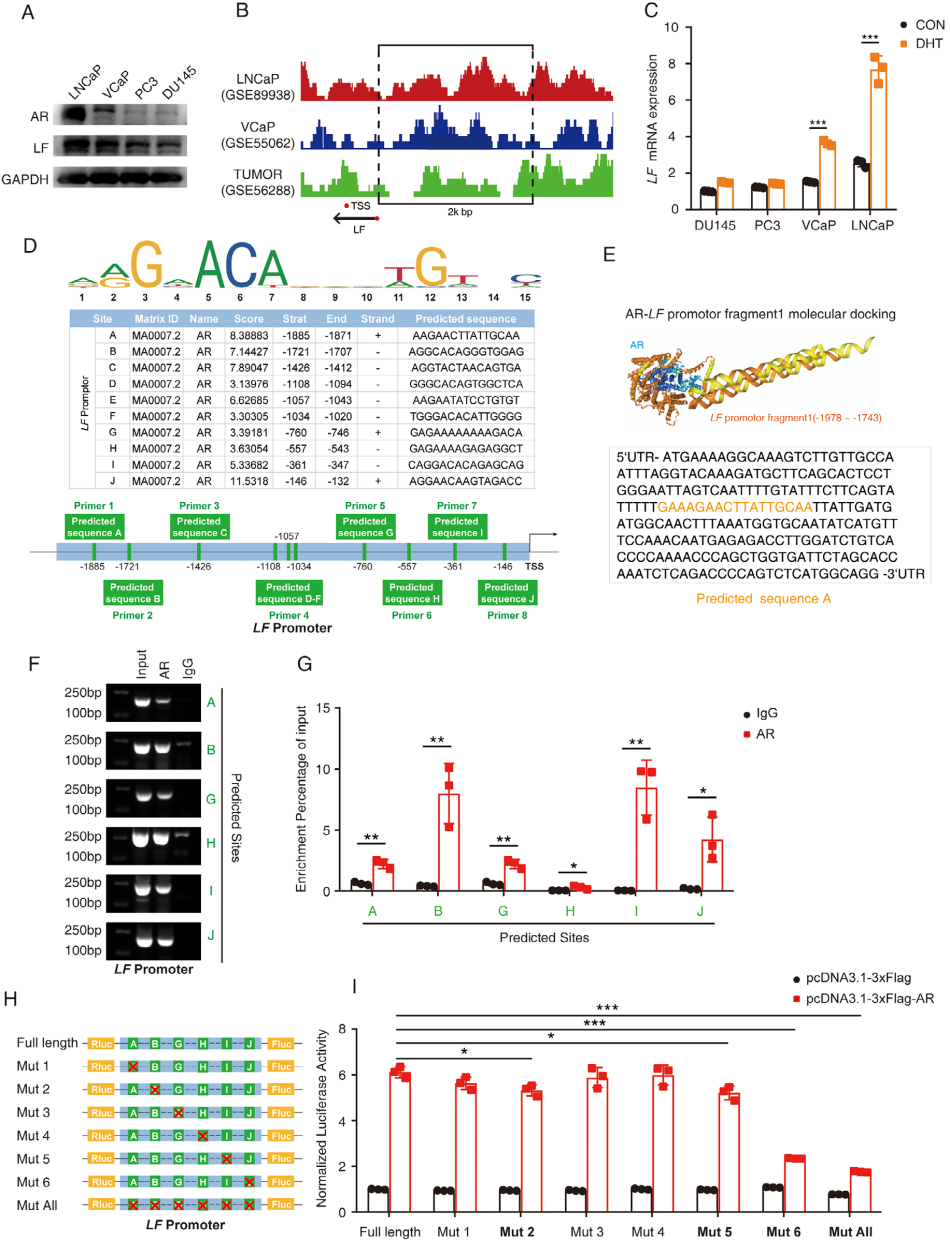
Androgen receptor (AR) transcriptionally regulates *LF* gene expression. (A) Protein levels of AR and LF in human prostate cancer cell lines: AR^+^ cells (LNCaP, VCaP) and AR^−^ cells (PC3, DU145). (B) AR ChIP‐Seq peaks on the upstream of the *LF* gene transcription start site (TSS) in LNCAP(GSE89938) cell lines, VCAP(GSE55062) cell lines, and human prostate tumor samples (GSE56288). (C) *LF* mRNA expression after DHT‐mediated AR activation in AR^+^ cells and AR^−^ prostate cancer cells. (D) Prediction of AR binding sites in the *LF* promoter region via JASPAR, including the AR binding motif, sequences of potential binding sites (A to J), their locations relative to the TSS, and primer design. (E) Molecular docking prediction of structural complexes between AR and *LF* promoter fragments 1(‐1978 to ‐1743, Predicted sequence A) via AlphaFold3. (F) ChIP‐PCR validation of AR binding to the *LF* promoter region. (G) ChIP‐qPCR quantification of AR binding enrichment at the *LF* promoter region. (H) Schematic of mutant dual‐luciferase reporter vectors. Rluc, *Renilla* luciferase; Fluc, *Firefly* luciferase. (I) Dual‐luciferase reporter assay evaluating AR transactivation on wild‐type and mutant *LF* promoters. *n* = 3, *p* < 0.05 “*,” *p* < 0.01 “**” (one‐way ANOVA for multi‐group comparisons; Student's *t*‐test for two‐group comparisons).

Lactoferrin demonstrates a close association with androgen response signaling (Figure 2G) and prostate epithelial cells (Figure 4). In prostate cancer cell lines, lactoferrin is highly expressed in AR‐positive cell lines (LNCaP and VCaP), but is lowly expressed in AR‐negative cell lines (PC3 and DU145) (Figure 5A). AR binds to the promoter of the *LF* gene (Figure 5B), as shown by ChIP‐Seq analysis [28, 29, 30]. Treatment with 10 nM dihydrotestosterone (DHT) to activate androgen receptors resulted in significantly increased *LF* gene expression in AR‐positive cell lines, while no substantial changes occurred in AR‐negative lines (Figure 5C). Meanwhile, treatment with enzalutamide (ENZ) in AR‐positive cell lines LNCaP and VCaP (1 µM) and *Lf* ^+/+^ TRAMP mice (20 mg/kg) significantly decreased *LF* gene expressions (Figure S4A,B).

Analysis of the *LF* gene promoter region (−2000 bp) using JASPAR revealed 10 potential AR binding sites, designated as “Predicted sequence A‐J” (Figure 5D) based on their distance from the transcriptional start site (TSS). AlphaFold3 structural modeling predicted specific AR‐DNA interactions across eight promoter fragments, divided by the potential AR binding sites identified by JASPAR in the *LF* gene promoter region, with results shown in Figure 5E and Figure S5 (Supporting Information). ChIP‐PCR/qPCR confirmed AR enrichment at the six predicted sites: A, B, G, H, I, J (Figure 5F,G, and Figure S4C). To pinpoint functional sites, we engineered dual‐luciferase reporters containing wild‐type *LF* promoters or mutated *LF* promoters (Figure 5H, and mutant sequence: Figure S4D). Through co‐expression of AR‐expressing vector and dual‐luciferase reporter vectors, significantly reduced luciferase activity was observed following mutation of sites B, I, and J (Mut2, Mut5, and Mut6), indicating that these three sites (B, I, and J) serve as the primary binding sites for transcription factor AR targeting the *LF* gene promoter region (Figure 5I).

### 2.6 Lactoferrin Inhibits Ferroptosis in Prostate Cancer Cells

**FIGURE 6:**
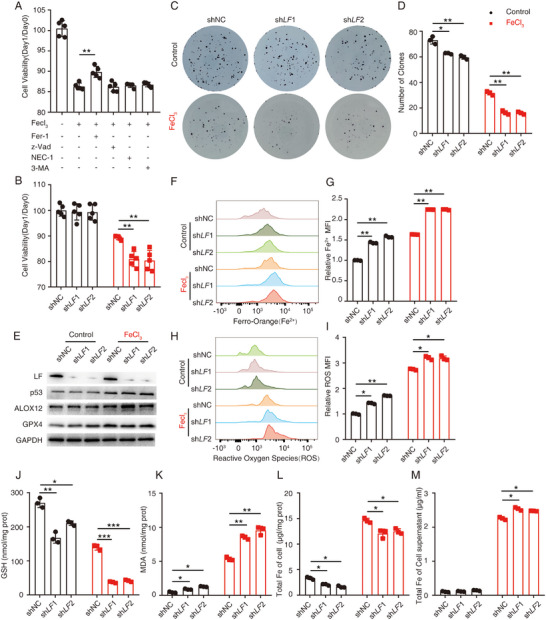
*LF* knockdown promotes ferroptosis in AR^+^ prostate cancer cells under iron overload. (A) Viability of LNCaP cells treated with 100 µM FeCl_3_ and different cell death inhibitors: Fer‐1 (ferroptosis inhibitor, 1 µM), z‐Vad (apoptosis inhibitor, 1 µM), Nec‐1 (necroptosis inhibitor, 1 µM), 3‐MA (autophagy inhibitor, 1 µM), *n *= 5. (B) Cell viability after *LF* knockdown via lentiviral vectors under 100 µM FeCl_3_, *n* = 5. (C) Colony formation capacity of *LF*‐knockdown (sh*LF*) LNCaP cells under iron stress (100 µM FeCl_3_), control: saline, shNC: non‐targeting shRNA. (D) Quantification of colony numbers, *n* = 3. (E) Protein expression of LF, p53, ALOX12, and GPX4 under iron stress. (F) Intracellular Fe^2^^+^ levels (FerroOrange staining) under iron stress. (G) Quantification of Fe^2^^+^ fluorescence intensity, *n* = 3. (H) Intracellular ROS levels (DHE probe) under iron stress. (I) Quantification of ROS fluorescence intensity, *n* = 3. (J) Glutathione (GSH) concentration under iron stress, *n* = 3. (K) Malondialdehyde (MDA) concentration under iron stress, *n* = 3. (L) Total intracellular iron concentration under iron stress, *n* = 3. (M) Total iron content in cell culture supernatant under iron stress, *n* = 3. *p* < 0.01 “**,” *p* < 0.001 “***” (one‐way ANOVA for multi‐group comparisons).

Since the prostate tumor tissue is more susceptible to ferroptosis in the *Lf* ^−/−^ TRAMP mice compared to *Lf* ^+/+^ TRAMP littermates, we then investigated the underlying mechanisms in prostate cancer cell lines with varying AR status. In LNCaP cells (AR^+^ prostate cancer cell line), the ferroptosis inhibitor Fer‐1 (1 µM) rescued Fe^3^^+^‐induced cytotoxicity (*p *< 0.001), whereas inhibitors targeting apoptosis (z‐VAD, 1 µM), necroptosis (Nec‐1, 1 µM), and autophagy (3‐MA, 1 µM) had no protective effect (Figure 6A). Under iron stress, *LF* knockdown (sh*LF*1/sh*LF*2) significantly reduced cell viability (Figure 6B) and clonogenic capacity (Figure 6C,D), and up‐regulated key ferroptotic regulators p53 and ALOX12 expressions (Figure 6E). The ferroptosis inhibitor Fer‐1 (1 µM) alleviated ferroptosis stress in *LF* knockdown (sh*LF*1/sh*LF*2) cells by reducing the expression of p53 and ALOX12 and upregulating SLC7A11 (Figure S6). Furthermore, *LF* knockdown resulted in elevated ferroptotic biomarkers, including intracellular Fe^2^^+^ (Figure 6F,G), ROS levels (Figure 6H,I), and MDA levels (Figure 6K), accompanied by a decline in GSH levels (Figure 6J), which also impaired cellular iron storage ability (Figure 6L,M). In DU145 cells (AR^−^ prostate cancer cell line), the ferroptosis inhibitor Fer‐1 (1 µM) also significantly rescued Fe^3^^+^‐induced cytotoxicity (Figure S7A). Under iron stress, *LF* overexpression enhanced cell viability (Figure S7B) and clonogenic capacity (Figure S7C,D), and down‐regulated key ferroptotic regulators p53 and ALOX12 (Figure S7E). Furthermore, *LF* overexpression resulted in reduced ferroptotic biomarkers, including intracellular Fe^2^^+^ (Figure S7F,G), ROS levels (Figure S7H,I), and MDA levels (Figure S7K), accompanied by elevated levels of GSH (Figure S7J), which also improved cellular iron storage ability (Figure S7L,M).

### 2.7 Lactoferrin Requires Its Iron‐Binding Domain to Attenuate Ferroptosis

**FIGURE 7:**
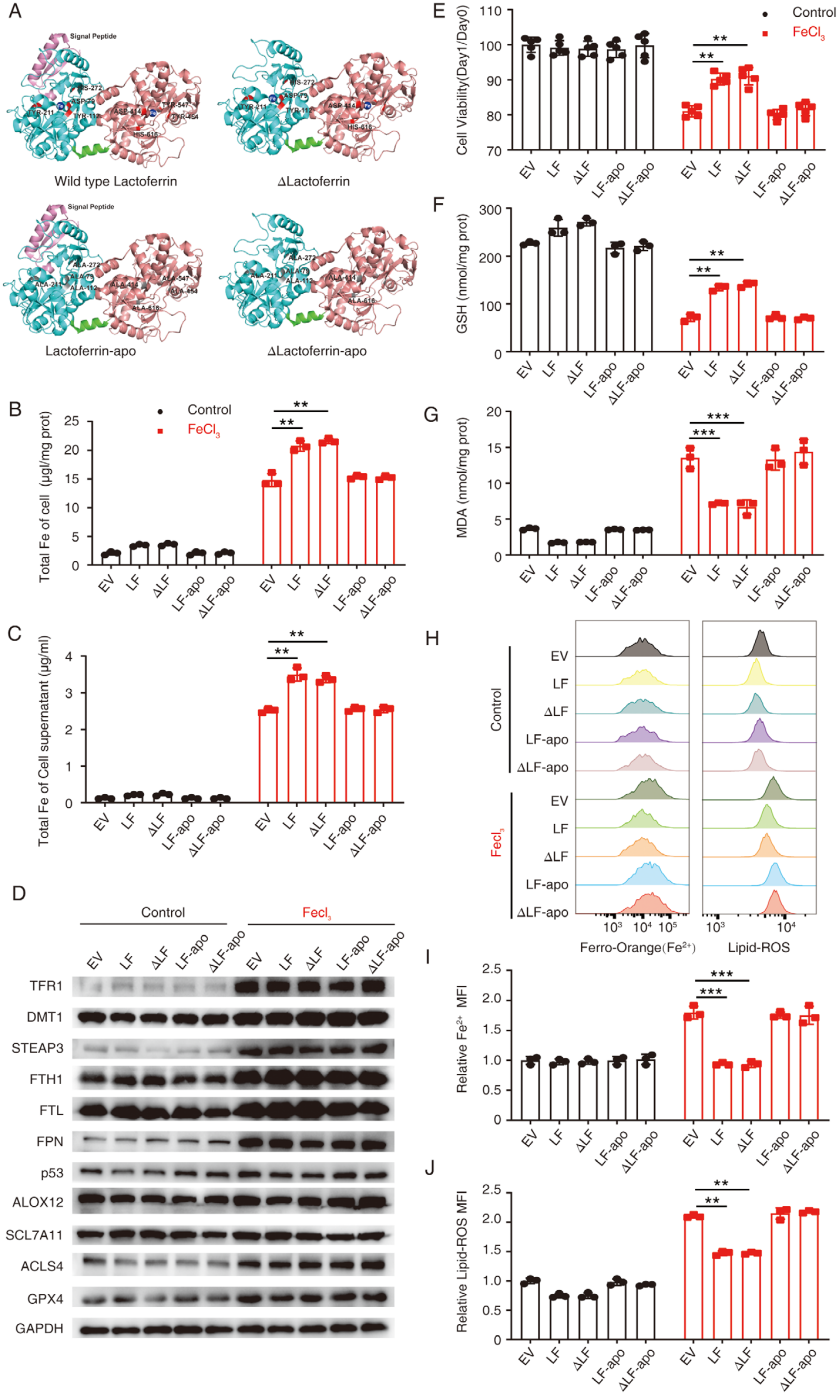
The iron‐binding domain of lactoferrin confers resistance to ferroptosis. (A) The schematics of three Lactoferrin mutant constructs: ΔLactoferrin (ΔLF, Δ1‐44, had the signal peptide deleted, causing lactoferrin to be localized intracellularly and unable to be secreted extracellularly); Lactoferrin‐apo (LF‐apo, iron‐binding‐deficient mutant, with alanine substitutions at eight key residues: Asp79, Tyr112, Tyr211, His272 in the N‐lobe, Asp414, Tyr454, Tyr547, His616 in the C‐lobe); ΔLactoferrin‐apo (ΔLF‐apo, double mutant combining both changes). (B) Total intracellular iron concentration under iron stress (100 µM FeCl_3_) in *LF*‐knockdown sh*LF*1‐LNCaP cells, control: saline, *n* = 3. (C) Total iron content in cell culture supernatant under iron stress, *n* = 3. (D) Proteins expression of iron metabolism (TFR1, DMT1, STEAP3, FTH1, FTL, and FPN) and ferroptosis (p53, ALOX12, SCL7A11, ACLS4, and GPX4) under iron stress. (E) Cell viability after sh*LF*1‐LNCaP cells under iron stress, *n* = 5. (F) Glutathione (GSH) concentration under iron stress, *n* = 3. (G) Malondialdehyde (MDA) concentration under iron stress, *n* = 3. (H) Intracellular Fe^2^^+^ levels (FerroOrange staining) and Lipid ROS levels (Liperfluo staining) under iron stress. (I) Quantification of Fe^2^^+^ fluorescence intensity, *n* = 3. (J) Quantification of Lipid ROS fluorescence intensity, *n* = 3. *p* < 0.001 “***” (one‐way ANOVA for multi‐group comparisons).

To identify the functional domain of lactoferrin responsible for attenuating ferroptosis, three *LF* mutants were generated: one vector (ΔLF) had the signal peptide deleted [31], causing lactoferrin to be localized intracellularly and unable to be secreted extracellularly; one vector (LF‐apo) had the amino acid residues responsible for iron ion binding mutated [6, 32], rendering lactoferrin incapable of binding iron ions; and one vector (ΔLF‐apo) had both of these mutations simultaneously (designs depicted in Figure 7A; validation data in Figure S8). Under iron stress, LF and ΔLF rescued cellular iron storage ability and up‐regulated the key iron storage protein ferritin (FTH1/FTL), and the canonical ferroptosis suppressor SLC7A11, while down‐regulating the ferroptotic regulators p53 and ALOX12. In contrast, LF‐apo and ΔLF‐apo lost lactoferrin's ability to modulate intracellular iron activity and had no significant effect on the expression of these proteins in sh*LF*1‐LNCaP cells (Figure 7B–D). Consistent with the functional requirement of the iron‐binding domain, the biochemical and phenotypic hallmarks of ferroptosis were differentially regulated by the lactoferrin variants. Overexpression of functional lactoferrin (LF and ΔLF) significantly increased cell viability (Figure 7E) and the level of GSH (Figure 7F), a key antiferroptotic antioxidant. Conversely, it markedly reduced the levels of pro‐ferroptotic indicators, including MDA (Figure 7G), intracellular Fe^2^^+^ (Figure 7H,I), and lipid ROS (Figure 7H,J). In contrast, the iron‐binding domain mutants (LF‐apo and ΔLF‐apo) failed to elicit these protective effects. These results demonstrate that lactoferrin exerts its anti‐ferroptotic function primarily through its iron‐binding domain.

### 2.8 Lactoferrin Deficiency Inhibits Iron‐Accelerated Prostate Cancer Progression

**FIGURE 8:**
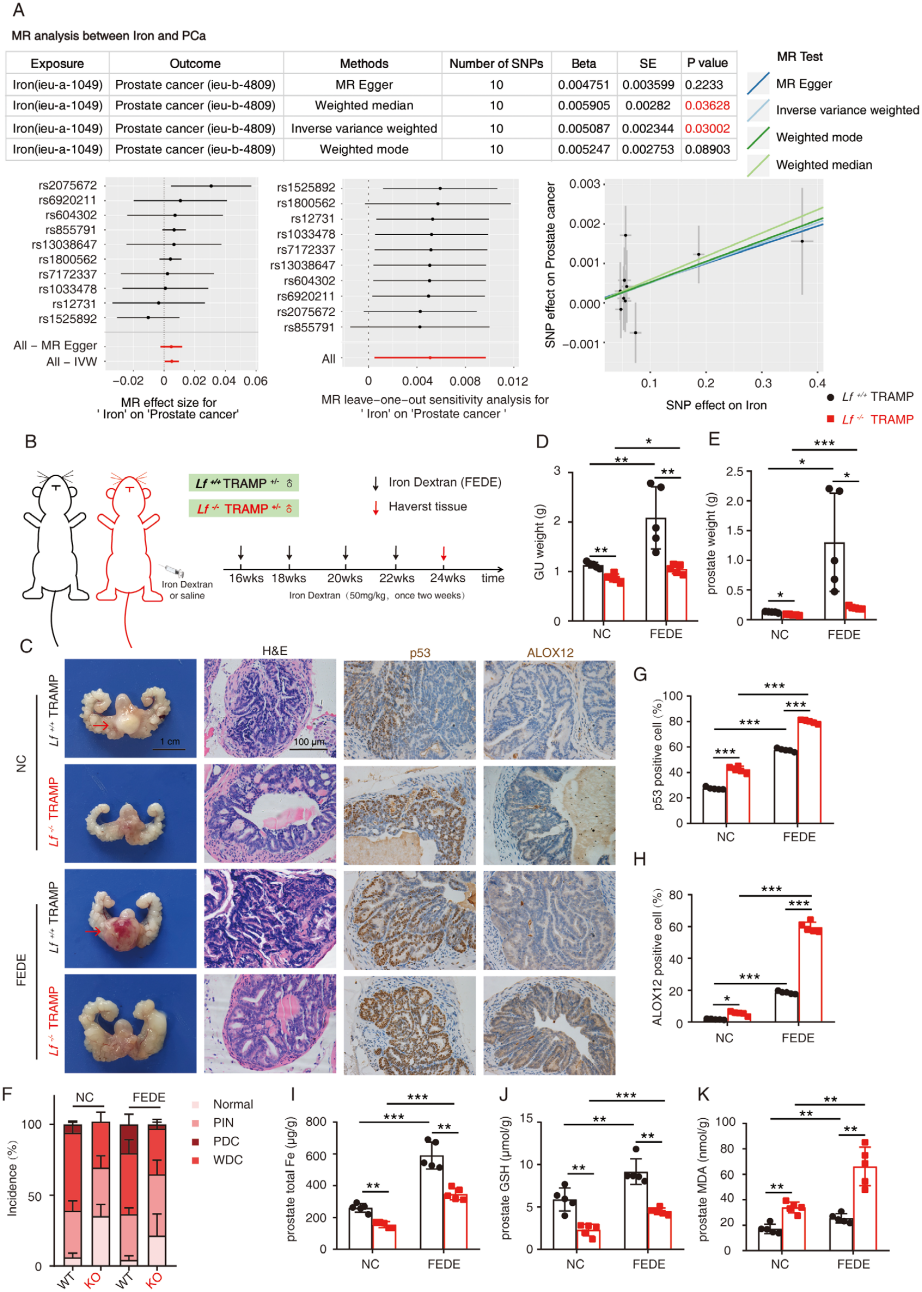
*LF* knockout inhibits iron‐accelerated prostate cancer progression. (A) Mendelian randomization analysis of iron status (ieu‐a‐1049) and prostate cancer risk (ieu‐b‐4809) using multivariable MR methods: MR‐Egger regression, Weighted median, Inverse‐variance weighted (IVW), and Weighted mode (MR‐Base platform). (B) Iron supplementation experimental design: (i) NC/TRAMP (ddH_2_O i.p. in *Lf* ^+/+^ TRAMP mice), (ii) NC/KO (ddH_2_O i.p. in *Lf* ^−/−^ TRAMP mice), (iii) FEDE/TRAMP (iron dextran 50 mg/kg i.p. in *Lf* ^+/+^ TRAMP mice), and (iv) FEDE/KO (iron dextran 50 mg/kg i.p. in *Lf* ^−/−^ TRAMP mice). (C) Pathological assessment with macroscopic GU morphology, H&E histopathology, and immunohistochemistry of p53 and ALOX12. (D) Quantification of GU weights. (E) Quantification of prostate tissue weights. (F) Pathological grading statistics: PDC (poorly differentiated adenocarcinoma), WDC (well‐differentiated adenocarcinoma), PIN (prostatic intraepithelial neoplasia), WT: *Lf ^+/+^* TRAMP, KO: *Lf ^‐/−^* TRAMP. (G) and (H) Percentage quantification of p53‐ and ALOX12‐ positive cells (%) in prostate tissues. (I) Iron ion levels in prostate tissue. (J) Glutathione (GSH) content in prostate tissues. (K) Malondialdehyde (MDA) content in prostate tissues. *n* = 5; *p* < 0.05 “*,” *p* < 0.01 “**,” *p* < 0.001 “***” (one‐way ANOVA for multi‐group comparisons).

After these findings in mouse models, we wanted to see what happened in clinical samples. Mendelian randomization analysis established a causal relationship (IVW: *p *= 0.030; Weighted Median: *p *= 0.036) between iron levels (ieu‐a‐1049) and human prostate cancer (ieu‐b‐4809) risk in the MR‐base website, prompting investigation of iron's paradoxical tumor‐promoting and ferroptosis‐inducing effects in TRAMP models (Figure 8A). Following this observation in the Mendelian randomization analysis, we divided the TRAMP mice into four groups: (i) NC/TRAMP (ddH_2_O i.p. in *Lf* ^+/+^ TRAMP mice), (ii) NC/KO (ddH_2_O i.p. in *Lf* ^−/−^ TRAMP mice), (iii) FEDE/TRAMP (iron dextran 50 mg/kg i.p. in *Lf* ^+/+^ TRAMP mice), and (iv) FEDE/KO (iron dextran 50 mg/kg i.p. in *Lf* ^−/−^ TRAMP) (Figure 8B). Iron dextran (FEDE) supplementation significantly accelerated tumor progression, as evidenced by increased genitourinary tract weight, prostate tissue weight, and histopathological scores in both *Lf* ^+/+^ TRAMP and *Lf* ^−/−^ TRAMP backgrounds; whereas lactoferrin deficiency (*Lf* ^−/−^ TRAMP) markedly attenuated these pro‐tumorigenic effects (Figure 8C–F). Concurrently, immunohistochemical staining revealed that the ferroptosis‐related proteins p53 and ALOX12, as well as AR and proliferation marker Ki67, were significantly upregulated in FEDE‐treated groups. Notably, lactoferrin deficiency enhanced the expression of p53 and ALOX12 while decreasing AR and Ki67 in prostate tissue (Figure 8C,G,H; and Figure S9). Furthermore, iron supplementation elevated key ferroptosis markers (total Fe, MDA, and decreased GSH) in both TRAMP groups. Besides, lactoferrin deficiency further increased the ferroptosis‐promoting factors MDA and Fe^2+^ while decreasing the ferroptosis‐inhibiting factor GSH (Figure 8I–K). Collectively, these results demonstrate the paradoxical effect of iron in prostate cancer mouse model—simultaneously driving tumor progression and ferroptotic stress—and establish lactoferrin as a key modulator of this balance, highlighting its therapeutic targetability.

### 2.9 Lactoferrin as a Proof‐of‐Concept Therapeutic Target in Prostate Cancer

**FIGURE 9:**
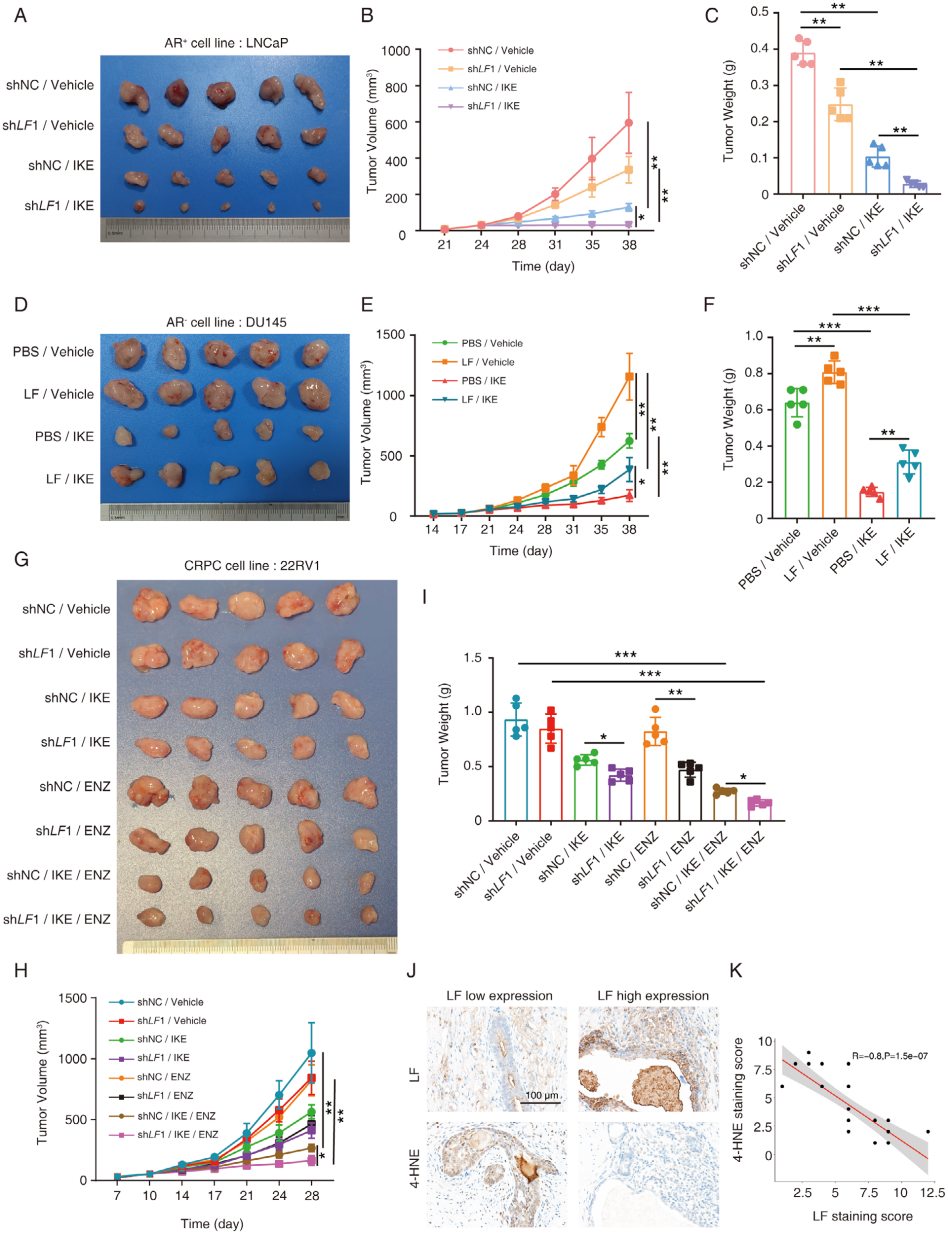
Preclinical targeting of lactoferrin sensitizes prostate cancer to ferroptosis induction. (A–C) AR^+^ LNCaP xenograft model with stable *LF* knockdown (sh*LF*1) and imidazole ketone erastin (IKE, 50 mg/kg) treatment: (i) shNC/Vehicle (non‐targeting shRNA, solvent i.p.); (ii) sh*LF*1/Vehicle (sh*LF*1, solvent i.p.); (iii) shNC/IKE (nontargeting shRNA, IKE i.p.); (iv) sh*LF*1/IKE (sh*LF*1, IKE i.p.). (A) photo of LNCaP xenograft. (B) Tumor growth curves of LNCaP xenograft. (C) Final tumor weights of LNCaP xenograft. (D‐F), AR^−^ DU145 xenograft model with LF (recombinant human lactoferrin, 50 mg/kg) and IKE (50 mg/kg) cotreatment: (i) PBS/Vehicle (PBS, solvent i.p.); (ii) LF /Vehicle (recombinant human lactoferrin, solvent i.p.); (iii) PBS/IKE (PBS, IKE i.p.); (iv) LF/IKE (recombinant human lactoferrin, IKE i.p.). (D) photo of DU145 xenograft. (E) Tumor growth curves of DU145 xenograft. (F) Final tumor weights of DU145 xenograft. (G–I) CRPC cell line 22RV1 xenograft model with stable *LF* knockdown (sh*LF*1), imidazole ketone erastin (IKE, 50 mg/kg), and enzalutamide (ENZ, 20 mg/kg) treatment: (i) shNC/Vehicle (nontargeting shRNA, solvent i.p.); (ii) sh*LF*1/Vehicle (sh*LF*1, solvent i.p.); (iii) shNC/IKE (nontargeting shRNA, IKE i.p.); (iv) sh*LF*1/IKE (sh*LF*1, IKE i.p.); (v) shNC/ENZ (non‐targeting shRNA, ENZ i.g.); (vi) sh*LF*1/ENZ (sh*LF*1, ENZ i.g.) (vii) shNC/IKE/ENZ (nontargeting shRNA, IKE i.p., ENZ i.g.) (viii) sh*LF*1/IKE/ENZ (sh*LF*1, IKE i.p., ENZ i.g.). (G) photo of 22RV1 xenograft. (H) Tumor growth curves of 22RV1 xenograft. (I) Final tumor weights of 22RV1 xenograft. (J) IHC staining of LF and 4‐HNE (lipid peroxidation marker) in 30 human prostate cancer specimens. (K) Correlation analysis between LF and 4‐HNE expression in 30 human prostate cancer specimens. (Spearman's *ρ* = ‐0.78, ^***^ *p* < 0.001). *n* = 5; *p* < 0.01 “**,” *p* < 0.001 “***” (one‐way ANOVA for multi‐group comparisons, two‐way ANOVA for tumor growth curves).

The potential of lactoferrin as a therapeutic target in prostate cancer was studied in the subcutaneous tumorigenicity of prostate cell lines (LNCaP: AR^+^; DU145: AR^−^, 22RV1: castration‐resistant prostate cancer (CRPC)). For the AR^+^ LNCaP xenografts, cells transduced with shNC or lactoferrin‐targeting shRNA (sh*LF*1) were stratified into: shNC‐Vehicle, sh*LF*1‐Vehicle, shNC‐IKE (50 mg/kg), and sh*LF*1‐IKE (50 mg/kg) groups (*n *= 5). By increasing ferroptotic stress, *Lf* knockdown (sh*LF*1) synergized with ferroptosis inducer IKE, reducing tumor volume vs. controls (Figure 9A–C, Figure S10A,B). For the AR^−^ DU145 xenografts, cells were assigned to: PBS‐Vehicle, LF (recombinant human lactoferrin, 50 mg/kg)‐Vehicle, PBS‐IKE (50 mg/kg), and LF (50 mg/kg)‐IKE (50 mg/kg) groups(*n *= 5). Recombinant LF protein promoted tumorigenicity and attenuated IKE efficacy (Figure 9D–F, Figure S10C,D). For the CRPC 22RV1 xenografts, cells transduced with shNC or sh*LF*1 were stratified into: shNC‐Vehicle, sh*LF*1‐Vehicle, shNC‐IKE (50 mg/kg), and sh*LF*1‐IKE (50 mg/kg) groups, shNC‐ENZ (20 mg/kg), sh*LF*1‐ENZ (20 mg/kg) groups, shNC‐IKE (50 mg/kg)‐ENZ (20 mg/kg), and sh*LF*1‐IKE (50 mg/kg)‐ENZ (20 mg/kg) groups (*n *= 5). *Lf* knockdown (sh*LF*1) synergized with ferroptosis inducer IKE and androgen receptor inhibitor ENZ, reducing tumor volume with increased ferroptotic stress vs. controls (Figure 9G–I, Figure S10E,F). Critically, immunohistochemical profiling of 30 clinical specimens revealed an inverse correlation between the expressions of lactoferrin and ferroptosis marker 4‐HNE (Spearman's *ρ* = −0.8, *p *< 0.001; Figure 9J,K). These results confirm lactoferrin‐mediated suppression of ferroptotic vulnerability in prostate tumors.

## 3 Discussion

Prostate cancer represents a major global health burden as the second most diagnosed malignancy in men worldwide [33]. Therefore, in‐depth exploration of the pathological characteristics and molecular mechanisms underlying prostate cancer pathogenesis is imperative. The current study revealed that lactoferrin is a previously unrecognized oncogene in prostate cancer, elucidating a novel AR‐lactoferrin‐ferroptosis regulatory axis with potential therapeutic prospects. Genetic ablation of *LF* in TRAMP mice significantly delayed tumor progression and enhanced ferroptosis pressure within prostate tissues. Mechanistically, the androgen receptor directly binds to the *LF* promoter, driving its transcription and subsequently suppressing the p53‐ALOX12 ferroptosis pathway. This protective mechanism against ferroptosis operates in both AR‐positive, AR‐negative, and AR inhibitor resistance contexts, suggesting lactoferrin's fundamental role in maintaining iron homeostasis in prostate cancer. This finding functionally resonates with the mechanism whereby the enhancer variant rs7077830 promotes ferroptosis and suppresses prostate cancer by regulating NCOA4 expression to facilitate ferritinophagy [34]. Together, these insights highlight the complexity and bidirectional regulatory potential of the ferroptosis and iron metabolism network in prostate cancer cells, which may jointly determine disease susceptibility and therapeutic response. The innovation of this study lies in establishing the core function of lactoferrin in prostate cancer: promoting tumor development by inhibiting ferroptosis, and clarifying its unique AR‐dependent regulatory mechanism. Our study highlights the context‐dependent role of lactoferrin in cancer. Contrary to its established tumor‐suppressive functions, we find that in prostate cancer, lactoferrin can exhibit oncogenic properties, a function that is mechanistically linked to and dependent on AR signaling.

The dual nature of iron metabolism in prostate cancer represents another layer of complexity in tumor biology. While iron serves as an essential metabolic cofactor supporting rapid tumor proliferation, it simultaneously poses a ferroptotic threat [35, 36, 37, 38]. Prostate tumorigenesis exhibits iron dependency, wherein elevated dietary iron intake correlates with increased cancer risk via Fenton reaction‐driven oxidative DNA damage [39]. Mechanistically, prostate tumor‐initiating cells (TICs) maintain expanded labile Fe^2^^+^ pools and demonstrate transcriptomic dysregulation of iron homeostasis genes (e.g., FPN↑, TFR1↑, HFE↑), collectively perturbing redox equilibrium to promote therapeutic resilience [40]. Consistent with this biochemical property, our analysis identified iron accumulation within prostate tumor tissues. Despite lactoferrin's canonical tumor‐suppressive functions in other malignancies, it undergoes a context‐dependent functional reversal in prostate cancer, where its iron‐buffering activity promotes carcinogenesis. This context‐dependent functionality raises two central questions: (i) Does the unique hormonal microenvironment of prostate tissue (e.g., androgen receptor signaling) reprogram lactoferrin's biological activity? (ii) Is lactoferrin's oncogenic role contingent upon iron saturation status, thereby modulating ferroptosis susceptibility? While iron fuels tumor metabolism, excess Fe^2+^ induces lethal ferroptosis. Lactoferrin reduces ferroptosis by sequestering labile iron, thereby inhibiting the p53‐ALOX12 ferroptosis execution axis and protecting cells from iron‐induced death. Critically, coordinate AR‐LF signaling establishes a cytoprotective program that buffers iron toxicity and sustains proliferation under oxidative stress, revealing a potential targetable vulnerability for advanced prostate cancer.

Our data demonstrated that iron supplementation in TRAMP mice accelerated tumor progression by enhancing proliferative signaling (Ki67, AR activation), while simultaneously inducing ferroptotic stress in prostate tissues. Crucially, *Lf* deficiency reduced both the tumor‐promoting effects and increased ferroptotic vulnerability. These findings support an emerging concept: the functional direction of lactoferrin in cancer is determined by cellular iron levels. Under iron stress, the AR‐hijacked lactoferrin acts as an iron “buffer” protecting cells; this context‐dependent role does not contradict but rather explains the pro‐ferroptotic effect of lactoferrin overexpression in other studies [41]. The unique oncogenicity exhibited by lactoferrin in prostate cancer contrasts sharply with its tumor‐suppressive role in other malignancies. The prostate is an androgen‐dependent organ, and the AR signaling pathway is the core oncogenic driver. Lactoferrin expression is directly driven by AR, leading to its “recruitment” into the oncogenic network, forcing its function to serve the survival needs of maintaining AR signaling rather than immune surveillance. Prostate cancer is an “iron‐loaded” cancer [42, 43]. In other tumors, lactoferrin may inhibit tumor growth by chelating iron ions; however, in prostate cancer, leveraging its exceptional iron‐binding capacity, lactoferrin acts as an iron ion “buffer,” maintaining iron levels at a precise balance point that meets the proliferation needs of cancer cells while avoiding triggering ferroptosis, thereby conferring a survival advantage. This iron‐dependent functional switching presents a promising therapeutic avenue. It further suggests that future ferroptosis‐targeted therapies may need to be personalized according to tumor iron status, guiding the strategic choice between inhibiting or co‐opting the lactoferrin pathway.

From a translational perspective, our findings provide strong preclinical proof‐of‐concept for targeting lactoferrin in both AR‐positive and AR‐negative prostate cancer contexts. In AR‐positive models (LNCaP and 22RV1), *LF* knockdown synergized remarkably with the ferroptosis inducer IKE in tumor growth suppression. Besides, in AR‐negative settings (DU145), recombinant lactoferrin protein effectively antagonized IKE‐mediated cytotoxicity. Notably, *LF* knockdown synergized remarkably with the ferroptosis inducer IKE and androgen receptor inhibitor ENZ in CRPC xenografts (22RV1). The clinical relevance of these findings is strengthened by the significant inverse correlation observed between lactoferrin and the ferroptosis marker 4‐HNE in human prostate cancer specimens. Targeting lactoferrin may offer an indirect yet highly efficient strategy: by degrading lactoferrin (e.g., using PROTAC technology), it is possible to attenuate the oncogenic output of AR signaling simultaneously and strongly sensitize cancer cells to ferroptosis inducers, achieving a highly efficient therapeutic effect.

Despite these advances, several limitations warrant further study. The precise molecular mechanisms connecting lactoferrin to p53‐ALOX12 regulation require further characterization. While our functional mutagenesis data suggest the importance of the iron‐binding domain, obtaining direct evidence of secretion or subcellular localization remains an interesting and valuable direction for future research to complement these findings. Though TRAMP models provide valuable insights, they incompletely recapitulate human prostate carcinogenesis; future studies on *Pten^−/−^* mouse models and patient‐derived organoids will address this limitation. To translate our preclinical proof‐of‐concept into a developed therapeutic strategy, these mechanistic and model‐related aspects require further exploration. It is important to note that the clinical associations observed in our cohort (*n* = 30), while supportive, remain preliminary and correlative. Therefore, future validation in larger clinical cohorts, functional studies in clinical specimens, and translational research are essential to establish the definitive utility of the LF‐ferroptosis axis as a biomarker or therapeutic guide. In conclusion, our work reveals lactoferrin as an androgen‐regulated guardian against ferroptosis in prostate cancer, providing a biochemical rationale for its context‐dependent functional duality. By disrupting the AR‐lactoferrin‐iron metabolic axis, we propose a novel potential therapeutic strategy to convert iron's pro‐tumor activities into a selective vulnerability.

## 4 Materials and Methods

### 4.1 Plasmids and Lentiviruses

The expression vector pIRES‐LF and pcDNA3.1‐3×FLAG‐AR and the luciferase reporter plasmids pDuaLuc‐*LF* promoter WT / Muts were purchased from YouBIO (Changsha, China). Lipofectamine 3000 transfection reagent (Thermo Fisher, USA) was used to transfect prostate cancer cells or HEK293T cells with plasmids. Lentiviruses for human *Lactoferrin* knockdown were purchased from HanBio (Shanghai, China), sequences followed as shNC: TTCTCCGAACGTGTCACGTAA, sh*LF*‐1: AAGACAGCCACGAACTCACTATTAT, and sh*LF*‐2: GGGACACTTCGTCCATTCTTGAATT. LNCaP cells were transduced with lentiviral particles at a multiplicity of infection (MOI) of 5 in the presence of 8 µg/mL polybrene (Sigma, USA). Stable clones were selected under continuous 2 µg/mL puromycin (Merck, Germany).

### 4.2 Reagents

Enzalutamide (ENZ), Imidazole ketone erastin (IKE), Ferrostatin‐1 (Fer‐1), Necrostatin‐1 (Nec‐1), Z‐VAD‐FMK (z‐Vad), 3‐Methyladenine (3‐MA), and Dihydrotestosterone (DHT) were purchased from Abmole (Shanghai, China); Iron Dextran (FEDE) was purchased from Macklin (Shanghai, China); FeCl_3_ and recombinant human lactoferrin were purchased from Sigma‐Aldrich.

### 4.3 Cell Lines

22RV1 (RRID: CVCL_1045), LNCaP (RRID: CVCL_0395), VCaP (RRID: CVCL_2235), PC3 (RRID: CVCL_C8XA), DU145 (RRID: CVCL_0105), and HEK293T (RRID: CVCL_0063) cell lines were purchased from the Cell Bank of the Chinese Academy of Sciences (Beijing, China). 22RV1, LNCaP, and DU145 cells were cultured in RPMI1640 cell culture medium (Gibco, USA) containing 10% FBS, 0.1% streptomycin, and 100 U/mL penicillin. VCap, PC3, and HEK293T cells were cultured in DMEM cell culture medium (Gibco, USA) containing 10% FBS, 0.1% streptomycin, and 100 U/mL penicillin. Cells were cultured in an incubator at 37°C and 5% CO_2_. All cell lines were authenticated by short tandem repeat (STR) profiling and tested negative for mycoplasma contamination before use.

### 4.4 Mouse Model

The *Lf* knockout mice (*Lf* ^−/−^) on a C57B/L6 background were generated as described previously [13, 14]. The transgenic adenocarcinoma of the mouse prostate (TRAMP) model mice [12a] were a kind gift from Professor Xiong Li at Guangdong Pharmaceutical University. After mating with *Lf* ^−/−^ TRAMP ^−/−^ and *Lf* ^+/+^ TRAMP ^+/−^ mice, the F1 progeny *Lf* ^+/−^ TRAMP ^+/−^ mice were generated. Then, through mating with *Lf* ^+/−^ TRAMP ^+/−^and *Lf* ^−/−^ TRAMP ^−/−^, the male offspring *Lf* ^−/−^ TRAMP ^+/−^ and *Lf* ^+/+^ TRAMP ^+/−^ mice were obtained (see Figure 1A for breeding scheme). Pathological progression was assessed at 8, 16, 24, and 36 weeks (*n *= 5) via the weight of the genitourinary system and prostate tissue, metastasis screening, and Kaplan–Meier survival analysis. For the iron supplementation test, 16‐week‐old littermates were randomized into four treatment groups: (i) NC/TRAMP (+ ddH_2_O i.p. in *Lf* ^+/+^ TRAMP mice), (ii) NC/KO (ddH_2_O i.p. in *Lf* ^−/−^ TRAMP mice), (iii) FEDE/TRAMP (iron dextran 50 mg/kg i.p. in *Lf* ^+/+^ TRAMP mice), and (iv) FEDE/KO (iron dextran 50 mg/kg i.p. in *Lf* ^−/−^ TRAMP mice) with biweekly injections until 24 weeks.

Male BALB/c nude male mice (4‐week‐old, *n *= 5) purchased from Hunan SJA Laboratory Animal Co., Ltd (Changsha, China) were subcutaneously inoculated with 2 × 10^6^ prostate cancer cells (LNCaP, DU145, or 22RV1) suspended in 100 µL Matrigel (Corning, USA): serum‐free medium (1:1). For the androgen receptor‐positive (AR^+^) LNCaP model, cells transduced with shNC or Lactoferrin‐targeting shRNA (sh*LF*1) were stratified into: shNC‐Vehicle (10% DMSO, 40% PEG300, and 5% Tween 80 in ddH_2_O), sh*LF*1‐Vehicle, shNC‐IKE (50 mg/kg), and sh*LF*1‐IKE groups. For the androgen receptor‐negative (AR^−^) DU145 model, cells were assigned to: PBS‐Vehicle, LF (recombinant human lactoferrin, 50 mg/kg)‐Vehicle, PBS‐IKE (50 mg/kg), and LF (50 mg/kg)‐IKE (50 mg/kg) groups. For the CRPC 22RV1 model, cells transduced with shNC or Lactoferrin‐targeting shRNA (sh*LF*1) were stratified into: shNC‐Vehicle, sh*LF*1‐Vehicle, shNC‐IKE (50 mg/kg), sh*LF*1‐IKE, shNC‐ENZ (20 mg/kg), sh*LF*1‐ENZ, shNC‐IKE‐ENZ, and sh*LF*1‐IKE‐ENZ groups. Tumor volume was measured twice a week. When tumors reached 100 ± 10 mm^3^, weekly intraperitoneal treatments (IKE) or oral gavage daily (ENZ) were administered. The animal studies were approved by the Animal Ethics Committee of Central South University (China).

### 4.5 Clinical Samples

Specimens from 30 histologically confirmed prostate cancer cases were obtained from the Second Xiangya Hospital of Central South University. All human tissue procurement procedures were approved by the Institutional Review Board of The Second Xiangya Hospital of Central South University, ensuring full compliance with the Declaration of Helsinki.

### 4.6 Quantitative Real‐Time PCR

Total RNA was isolated from cells using TRIzol reagent (Invitrogen, USA) according to the manufacturer's instructions, followed by the synthesis of cDNA through a Revert Aid First Strand cDNA Synthesis Kit (Thermo Fisher, USA). The primers sequence is as follows: *LF*: forward 5’‐GAGAAGGAGTGTTCAGTGGT‐3’, reverse 5’‐ATAGTGAGTTCGTGGCTGTC‐3’; *GAPDH*: forward 5’‐ATGACATCAAGAAGGTGGTG‐3’, reverse 5’‐CATACCAGGAAATGAGCTTG‐3’. The relative target gene mRNA levels were quantified by measuring the cycle threshold (Ct) values and then normalized to the *GAPDH* gene.

### 4.7 Western Blot

Total cellular proteins were extracted by ice‐cold RIPA lysis buffer (Beyotime, Shanghai, China) with 1× protease inhibitor cocktail (Bimake, USA) and phosphatase inhibitor cocktail (Bimake, USA). After separation by SDS‐PAGE gel, proteins were subsequently transferred to PVDF membranes (Millipore, USA) and blocked with 5% skim milk. Primary antibody incubations were performed overnight at 4°C, and the corresponding secondary antibody was incubated at 37°C. Signal detection was performed by an enhanced chemiluminescence (ECL) kit (Millipore, USA). The primary antibody list is shown in Table S1.

### 4.8 Enzyme Linked Immunosorbent Assay (ELISA)

The concentration of lactoferrin in the cell culture supernatant under iron stress was measured by the commercial ELISA kits according to the manufacturer's protocols (ELK Biotechnology, Wuhan, China).

### 4.9 Comprehensive Histological Analysis

After being fixed in fresh 4% paraformaldehyde for 24 h, tissues were dehydrated, paraffin‐embedded, and sectioned into 4 µm‐thick sections. Sections underwent deparaffinization in xylene and rehydration through a graded ethanol series (100%–50%). For H&E Staining, slides were stained with hematoxylin (3 min), differentiated (1% acid ethanol, 10 s), blued (slow‐flowing tap water), and counterstained with eosin (1 min). In dual‐iron valence histochemical Staining, tissue sections were stained with Turnbull's blue reagent (Bioss, Beijing, China) for 30 min at room temperature, forming characteristic blue Fe^2^^+^‐ferrocyanide complexes (Turnbull's blue). For ferric ion (Fe^3^^+^) detection, parallel sections were stained with Prussian blue reagent (Bioss, Beijing, China) under identical conditions, generating Fe^3^^+^‐ferricyanide deposits (Prussian blue). All slides underwent nuclear counterstaining with Nuclear Fast Red for 5 min at room temperature. For Immunohistochemistry (IHC), antigen retrieval in citrate buffer preceded peroxidase blocking (3% H_2_O_2_, 30 min). Primary antibodies were incubated overnight at 4°C, detected by HRP‐conjugated secondary antibody with DAB development. For immunofluorescence (IF), antigen‐retrieved sections were blocked with 5% goat serum, incubated with primary antibodies (4°C, overnight), then with Fluorescently conjugated secondary antibodies in the dark. Nuclei were counterstained with DAPI (Yeasen, Shanghai, China). All assessments were performed by two blinded pathologists. The primary antibody list is shown in Table S2.

### 4.10 Transmission Electron Microscopy

Murine prostate tissues (1 mm^3^) underwent primary fixation in 2.5% glutaraldehyde (4°C in 0.1 M phosphate buffer, overnight), followed by secondary fixation in 1% osmium tetroxide (4°C, 1 h). Samples were dehydrated through acetone gradients (30%→100%, 45 min/step), infiltrated with SPI‐PON 812 resin (30%→70%→pure, 1–5 h), and polymerized at graded temperatures (37°C→60°C, 48 h). Ultrathin sections (50–60 nm) were double‐stained with 3% uranyl acetate (30 min, RT) and Reynolds' lead citrate (8 min, RT with CO_2_ trap), then imaged on JEOL JEM‐1011 at 80 kV (10 000–50 000× magnification).

### 4.11 AlphaFold3‐Based Molecular Docking Prediction

The *LF* promoter sequence was segmented into eight fragments based on putative AR‐binding sites predicted by JASPAR (MA0007.2) based on their distance from the transcriptional start site (TSS): Fragment 1 (‐1978 to ‐1743), Fragment 2 (‐1800 to ‐1567), Fragment 3 (‐1566 to ‐1273), Fragment 4 (‐1152 to ‐862), Fragment 5 (‐855 to ‐623), Fragment 6 (‐681 to ‐390), Fragment 7 (‐447 to ‐219), and Fragment 8 (‐275 to ‐48). The full‐length androgen receptor (AR) amino acid sequence (UniProt ID: P10275, Homo sapiens) was retrieved from UniProt [44]. Protein‐DNA docking simulations were performed using AlphaFold3 Multimer v3.0 (https://alphafoldserver.com/) with default parameters [45].

### 4.12 Chromatin Immunoprecipitation

Chromatin immunoprecipitation (ChIP) was performed using the Magnetic ChIP Kit (Thermo Fisher, USA). In brief, LNCaP cells were crosslinked with 1% formaldehyde for 10 min at room temperature. Crosslinking was quenched with 125 mM glycine and then sonicated to 200–500 bp fragments at 4°C. For each immunoprecipitation,50 µg sheared chromatin was incubated overnight at 4°C with rotation in 5 µg anti‐AR antibody (5153S, CST, USA) or IgG control. Immune complexes were captured with 25 µL preblocked Protein A/G Magnetic Beads (2 h, 4°C) and reversed using 10 µg/mL Proteinase K (2 h, 65°C). DNA was purified for PCR and qPCR tests; the primer sequences are listed in Table S3.

### 4.13 Dual‐Luciferase Reporter Assay

The assay was using the Dual Luciferase Reporter Assay Kit (Vazyme, Nanjing, China) according to the manufacturer's instructions. HEK293T cells were seeded in 24‐well plates and transfected at 50% confluency using Lipofectamine 3000 (Thermo Fisher, USA). Each well received 0.5 µg pcDNA3.1‐3×Flag‐AR (OE plasmid) + 0.5 µg pDualLuc‐*LF* promoter (WT or Mutant) with technical duplicates. After 48 h, cells were washed twice with PBS and lysed in 100 µL 1× Cell Lysis Buffer for 5 min at room temperature. Lysate supernatants were assayed for *Firefly* luciferase activity and *Renilla* luciferase activity. Relative *Luciferase* Activity = *Firefly* Luciferase Activity/*Renilla* Luciferase Activity.

### 4.14 Intracellular Fe^2^^+^, ROS, and lipid ROS Detection

LNCaP and DU145 cells were seeded at 1 × 10^6^ cells/well in 6‐well plates and cultured overnight for adherence. The next day, cells were treated with 100 µM FeCl_3_ or saline vehicle control for 24 h. After being harvested with trypsin/EDTA (0.25%), cells were incubated with 1 µM FerroOrange (Dojindo, Japan) in Fe^2^^+^ detection, 10 µM dihydroethidium (Boxbio, Beijing, China) in ROS detection, or 1 µM Liperfluo (Solarbio, Beijing, China) in ROS detection for 30 min at 37°C in darkness. Stained cells were filtered and analyzed via flow cytometry.

### 4.15 Cell Viability Assay

LNCaP and DU145 cells were seeded at 1000 cells/well into 96‐well plates and cultured overnight for adherence. Cells were treated with 100 µM FeCl_3_ combined with the following cell death inhibitors for 24 h: Fer‐1 (Ferroptosis inhibitor, 1 µM), Nec‐1 (Necroptosis inhibitor, 1 µM), z‐Vad (Apoptosis inhibitor, 1 µM), and 3‐MA (Autophagy inhibitor, 1 µM) for 24 h. Viability was assessed using CCK‐8 reagent (APExBIO, USA) at 450 nm according to the manufacturer's instructions.

### 4.16 Colony Formation Assay

LNCaP and DU145 cells were seeded at 1000 cells/well into 6‐well plates and cultured overnight for adherence. The next day, cells were treated with 100 µM FeCl_3_ or saline vehicle control, with medium containing treatments replaced every 72 h during a 9‐day culture. Colonies were fixed with methanol, stained with 0.01% crystal violet for 1 h, and destained with water.

### 4.17 Biochemical Experiments

The preparation of serum, cell lysates, and tissue supernatants was according to the manufacturer's instructions. The concentration of total iron was measured by the Iron tissue iron assay kit (Jianchengbio, Nanjing, China) at 520 nm. The concentration of Glutathione (GSH) was measured by the Reduced glutathione assay kit (Jianchengbio, Nanjing, China) at 405 nm. And the concentration of Malondialdehyde (MDA) was measured by Malondialdehyde content assay kit (Boxbio, Beijing, China) at 532/450/600 nm.

### 4.18 Mendelian Randomization Analysis

Two‐sample MR analysis was employed by the MR‐Base platform (http://app.mrbase.org/) to investigate causal effects of systemic iron (exposure: ieu‐a‐1049) status on prostate cancer (outcome: ieu‐b‐4809) risk [46]. The analysis included: Inverse‐variance weighted (IVW) as the primary method; Weighted median for robustness validation; MR‐Egger regression to assess horizontal pleiotropy; Weighted mode for heterogeneous effect estimation.

### 4.19 TMT‐Based Quantitative Proteomics

Proteins extracted from 36 weeks *Lf* ^−/−^ TRAMP and *Lf* ^+/+^ TRAMP mice prostate tissues (*n *= 2) were trypsin‐digested (37°C, 16 h) and labeled with TMTpro 16plex reagents (Thermo Fisher, USA). LC‐MS/MS Sequencing by OE Biotech Co., Ltd. (Shanghai, China). Labeled peptides were analyzed via Orbitrap Fusion Lumos with 120‐min gradients (3%–28% ACN/0.1% FA). MS parameters included: 120K MS1 resolution (350–1550 m/z), HCD fragmentation (NCE 38%), and MS2 at 50K resolution. Data processing used MaxQuant v2.0.3 against UniProt Mouse (UP000000589) with 1% FDR threshold. The mass spectrometry proteomics data have been deposited to the ProteomeXchange Consortium (https://proteomecentral.proteomexchange.org) via the iProX partner repository with the dataset identifier PXD067106 [47, 48].

### 4.20 Bioinformatic Data Acquisition

Transcriptomic profiles were integrated from: TCGA‐PRAD: mRNA expression (HTSeq‐FPKM) for 499 prostate adenocarcinoma samples (GDC portal, v32.0), processed via R/Bioconductor (DESeq2 normalization, log_2_(FPKM+1) transformation) and GTEx v8: Normal prostate tissue expression (*n *= 100, UCSC Xena) co‐normalized with TCGA using ComBat batch correction. Weighted Gene Co‐expression Network Analysis (WGCNA) was performed on the TCGA‐PRAD dataset. To ensure network robustness, only the top 25% of genes with the highest variance were included. A soft‐thresholding power of beta = 4 was selected to construct a scale‐free topology network (R^2^> 0.85). Gene modules were identified using the dynamic tree cut algorithm with a minimum module size of 30 and a merge cut height of 0.25. To identify biologically significant modules, Pearson correlation analysis was conducted between module eigengenes (MEs) and clinical/molecular traits, including LF expression levels and pathway activity scores for Ferroptosis (GPX4, SLC7A11, ACSL4, ACSL3, TFRC, FTH1, FTL, TP53, NCOA4, ALOX12, HMOX1) and AR signaling (MYC, FKBP4, SERBP1, AR, NKX3‐1, CCND1, TMPRSS2, CDKN1A, SRD5A2), which were pre‐quantified using the ssGSEA algorithm. The WGCNA network was imported into Cytoscape for visualization. Bulk RNA‐seq GSE6919 [49] and scRNA‐seq GSE193337 [50] were downloaded from the Gene Expression Omnibus (GEO, https://www.ncbi.nlm.nih.gov/geo/). The FFPE_Bx cohort data were sourced from the GEO dataset GSE220095 [20]. The MSKCC_2010 cohort data were sourced from GSE21032 [21]. The PRAD_FHCRC cohort data were sourced from GSE74685 [22]. The GSE89938 [28], GSE55062 [29], and GSE56288 [30] were performed on AR‐binding genes ChIP‐Seq analysis.

### 4.21 Functional Enrichment Analysis

The differently expressed proteins between *Lf* ^−/−^ TRAMP and *Lf* ^+/+^ TRAMP group or genes between 25% *LF* high expression PRAD and 25% low *LF* expression PRAD group were selected with a threshold of *p* < 0.05 and fold change> 1.2 or <0.8. The upregulated proteins in the *Lf* ^+/+^ TRAMP group or 25% *LF* high expression PRAD were analyzed with GO enrichment analysis and KEGG pathway analysis by DAVID Bioinformatics Resources 6.8 (https://david.ncifcrf.gov/). Gene Set Enrichment Analysis (GSEA, https://www.gsea‐msigdb.org/gsea/index.jsp) was used to compare the difference in gene expression landscape between proteomic and transcriptomic profiles.

### 4.22 Statistical Analysis

All data were analyzed using SPSS 22.0, GraphPad Prism 5, and R 4.0.2. Continuous variables were assessed with: Student's *t*‐test for two‐group comparisons; One‐way ANOVA for multi‐group comparisons; Spearman's rank correlation for immunohistochemical scoring in prostate cancer tissues; and Survival analysis employed the Kaplan‐Meier method with the log‐rank test. The differences were considered statistically significant if *p* < 0.05, and *p* < 0.05 is indicated by “*,” *p *< 0.01 is indicated by “**,” *p* < 0.001 is indicated by “***.”

## Author Contributions

C.L., Q.P., X. Z., Z. L., Y. Wu., Y. Wen., R. Z., S.W., L. S., X. M., and Q.L. collected, analyzed, and interpreted data; Y. M., C. O, C. Z., and Z.H. performed bioinformatic and computational analysis; C.L., M. Z., S.F., L.W., and J. M. analyzed the results and wrote the manuscript; C.L., L.W. and J. M. conceived the project.

## Conflicts of Interest

The authors declare no conflicts of interest.

## Supporting information




**Supporting File**: advs75203‐sup‐0001‐SuppMat.pdf

## Data Availability

The data that support the findings of this study are available from the corresponding author upon reasonable request.
