# Supplementary material for: Androgen Receptor‐Induced Lactoferrin Accelerates Prostate Tumorigenesis Through Modulating Ferroptosis
Source: Adv Sci (Weinh). 2026 Apr 7:e20109. Online ahead of print. doi: 10.1002/advs.202520109 (PMC13334613; doi:10.1002/advs.202520109)
Supplement: Supplementary file 1 — Supporting File: advs75203‐sup‐0001‐SuppMat.pdf [file ADVS-9999-e20109-s001.pdf]

ADVANCED SCIENCE

Androgen receptor-induced lactoferrin accelerates prostate tumorigenesis through modulating ferroptosis

Liu et al.

Supporting Information

Supplementary Figures (1-10)

Supplementary Table 1. Primary antibodies used for Western Blot

Supplementary Table 2. Primary antibodies used for Immunohistochemistry and Immunofluorescence

Supplementary Table 3. Primers used for ChIP-PCR/qPCR

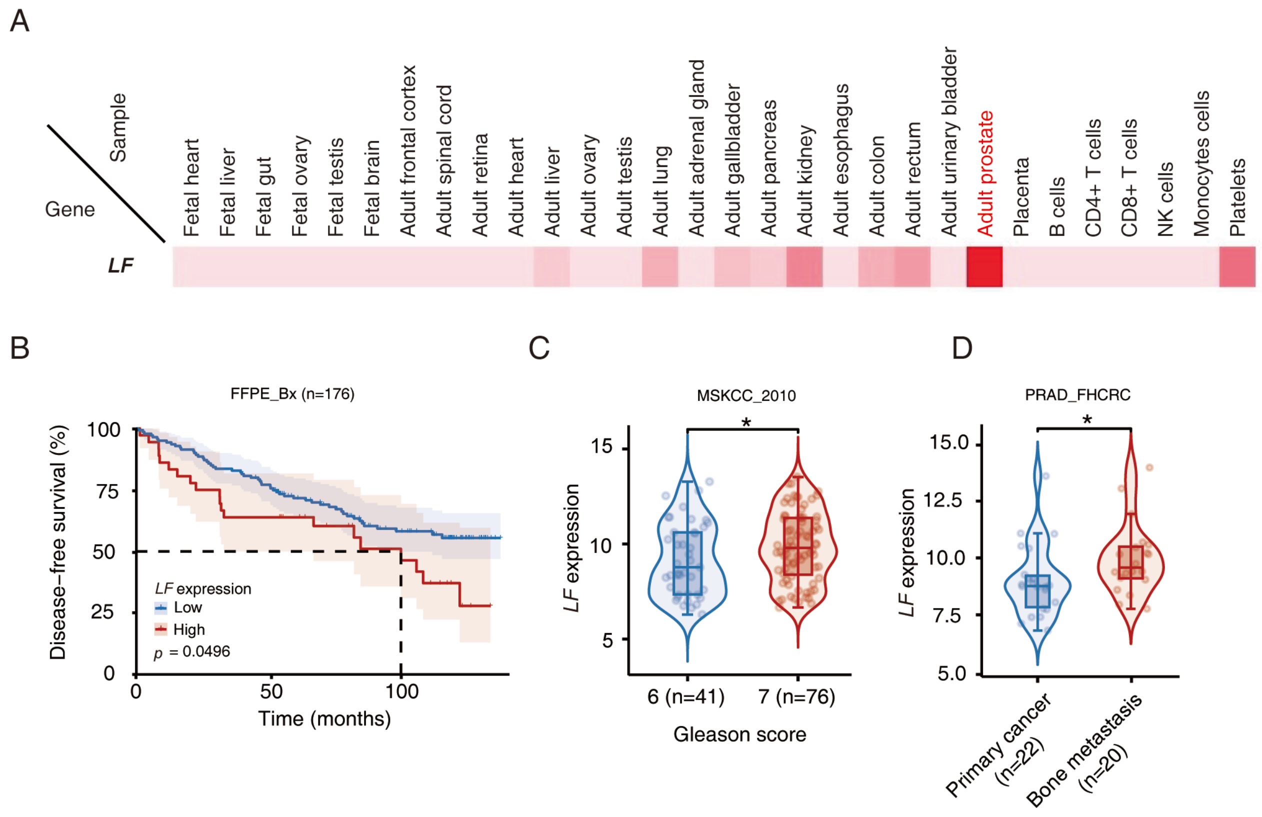

Figure S1 (related to Figure 1). Elevated *LF* expression is associated with aggressive disease progression in prostate cancer. (A) LF expression abundance across human tissues (The Human Protein Atlas 1101 database). (B) High *LF* expression is associated with worse disease-free survival (GSE220095). (C) *LF* expression positively correlates with Gleason score of prostate cancer patients (GSE21032). (D) *LF* expression is significantly upregulated in bone metastases compared to primary tumor tissues of prostate cancer patients (GSE74685).

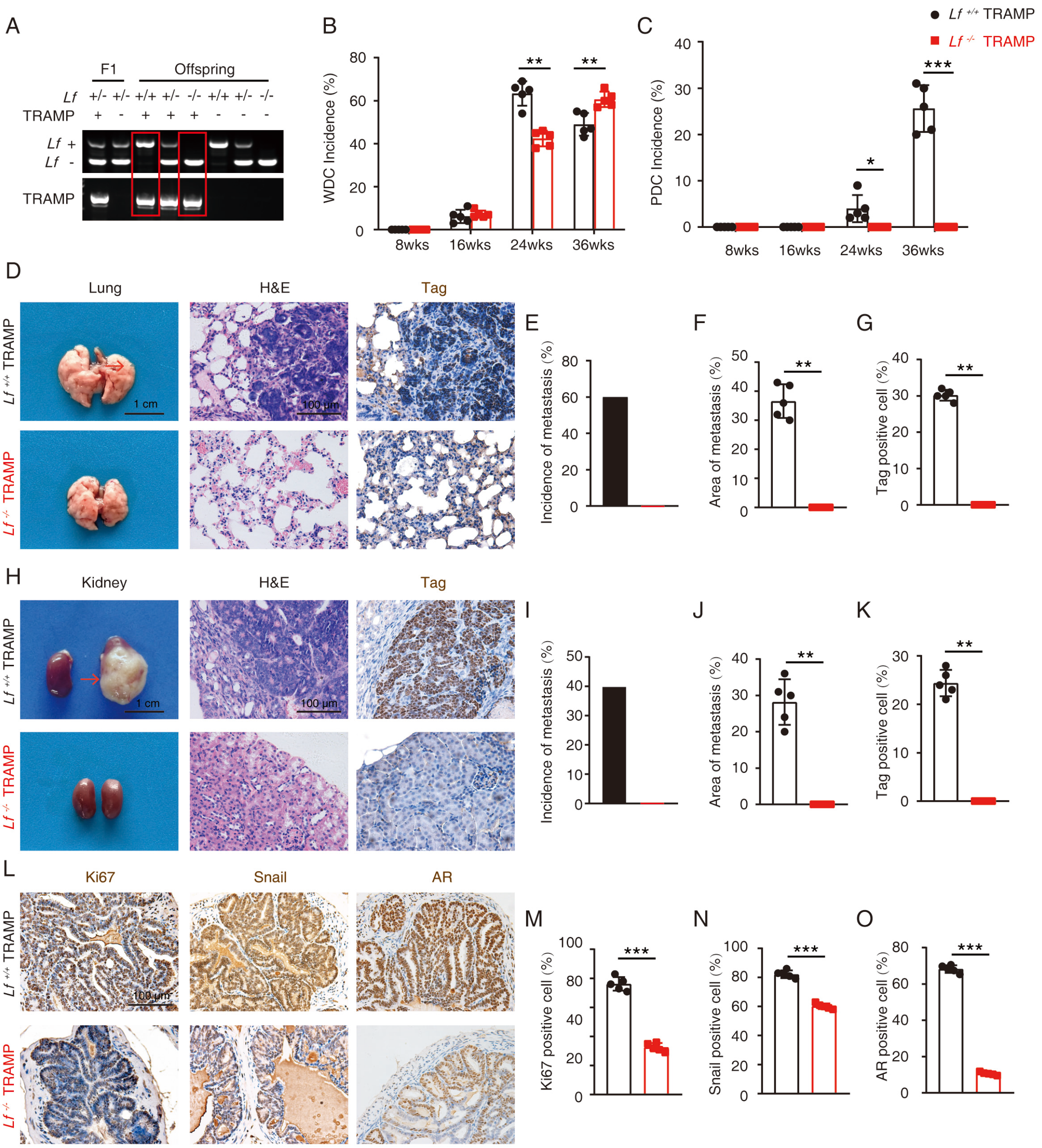

Figure S2 (related to Figure 1). Lactoferrin deficiency suppresses prostate cancer proliferation and metastasis in TRAMP mice. (A) Genotypic validation of *Lf* knockout TRAMP mice, Red boxes indicate target genotypes ($Lf^{+/+}$ TRAMP and $Lf^{-/-}$ TRAMP). (B) and (C) Pathological grading statistics of PDC (poorly differentiated adenocarcinoma) and WDC (well-differentiated adenocarcinoma) at 8, 16, 24, and 36 weeks. (D) Pulmonary metastases in $Lf^{+/+}$ TRAMP and $Lf^{-/-}$ TRAMP mice at 36 weeks. (E) Metastatic incidence rate in lung tissues. (F) Percentage of metastatic area in lungs. (G) Quantification of T-antigen (Tag)-positive cells in pulmonary metastases. (H) Renal metastases in $Lf^{+/+}$ TRAMP and $Lf^{-/-}$ TRAMP mice at 36 weeks. (I) Metastatic incidence rate in kidney tissues. (J) Percentage of metastatic area in kidneys. (K) Quantification of Tag-positive cells in renal metastases. (L) Immunohistochemical staining of Ki67 (proliferation), Snail (metastasis), and AR (androgen receptor) in prostate tissues. (M-O) Percentage quantification of Ki67-, Snail-, and AR-positive cells in prostate tissues. $n = 5$, $p < 0.01$ "**", $p < 0.001$ "***" (Student's *t*-test).

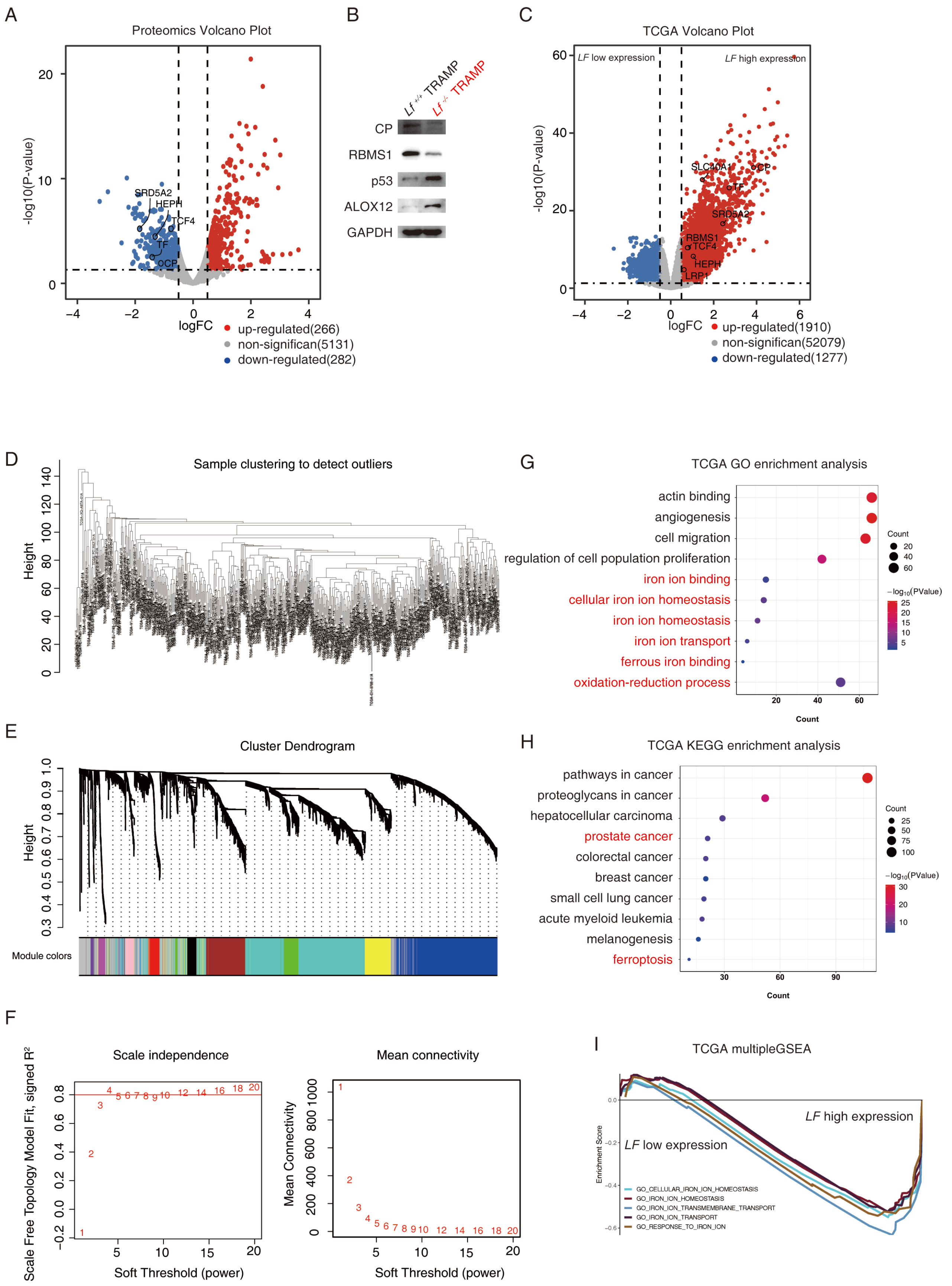

Figure S3 (related to Figure 2). Supplementary analysis of lactoferrin-associated dysregulated signaling pathways. (A) Volcano plot of differentially expressed proteins in prostate tissues from

Lf$^{-/-}$ TRAMP vs. *Lf*$^{+/+}$ TRAMP mice (n = 2). (B) Protein expression of CP, RBMS1, p53, and ALOX12 in TRAMP mice (*Lf*$^{-/-}$ vs. *Lf*$^{+/+}$) prostate tissue by means of Western blotting. (C) Volcano plot of differentially expressed genes (DEGs) between *LF*-high (top 25%) and *LF*-low (bottom 25%) groups in TCGA-PRAD prostate cancer patient cohort. (D) Hierarchical clustering of TCGA-PRAD samples based on gene expression profiles to identify potential outliers prior to network construction. (E) Hierarchical clustering dendrogram of genes based on topological overlap, with co-expression modules indicated by distinct colors. (F) Scale-free topology model fit (left) and mean connectivity (right) across different soft-thresholding powers. The selected power achieves an approximate scale-free topology while preserving sufficient network connectivity. (G) GO enrichment of upregulated DEGs in *LF*-high TCGA-PRAD samples. (H) KEGG pathway enrichment of upregulated DEGs in *LF*-high TCGA-PRAD samples. (I) Multivariate GSEA of transcriptomic profiles comparing *LF*-low vs. *LF*-high groups (TCGA-PRAD).

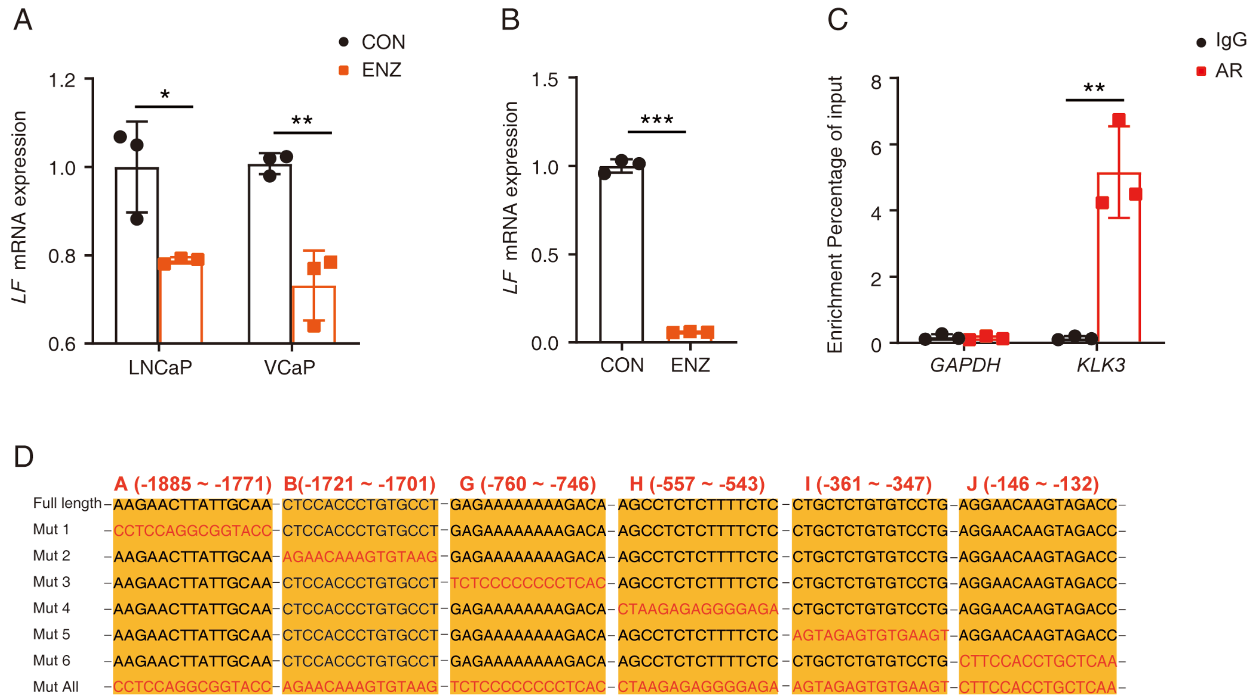

Figure S4 (related to Figure 5). AR directly activates lactoferrin transcription. (A) *LF* mRNA expression after ENZ-mediated AR inhibition in AR^{+} prostate cancer cells (LNCaP, VCaP, 1 μM). (B) *LF* mRNA expression after ENZ-mediated AR inhibition in TRAMP mice prostate tissue (20 mg/kg). (C) Negative (GAPDH) and positive (KLK3) controls for ChIP-qPCR. (D) Mutagenesis design for dual-luciferase reporter assays in *LF* promoter regions.

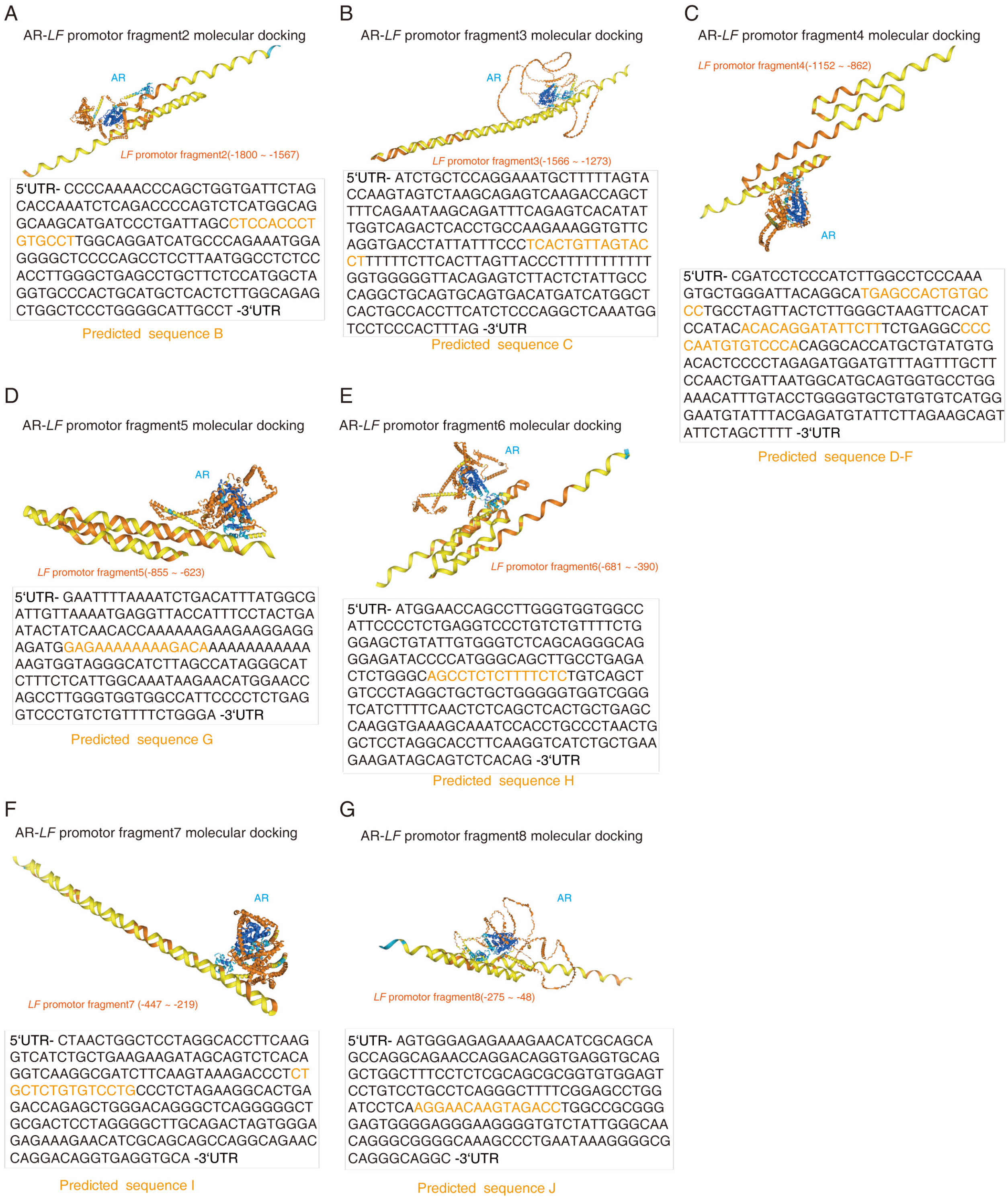

Figure S5 (related to Figure 5). AlphaFold3-predicted structural basis of AR binding to *LF* promoter fragments (2-8). (A-G) Predicted complex structures between the AR DNA-binding domain and *LF* promoter fragments. (A) Fragment 2 (-1800 to -1567): Sequence B. (B) Fragment 3 (-1566 to -1273): Sequence C. (C) Fragment 4 (-1152 to -862): Sequences D-F. (D) Fragment 5 (-855 to -623): Sequence G. (E) Fragment 6 (-681 to -390): Sequence H. (F) Fragment 7 (-447 to -219): Sequence I. (G) Fragment 8 (-275 to -48): Sequence J.

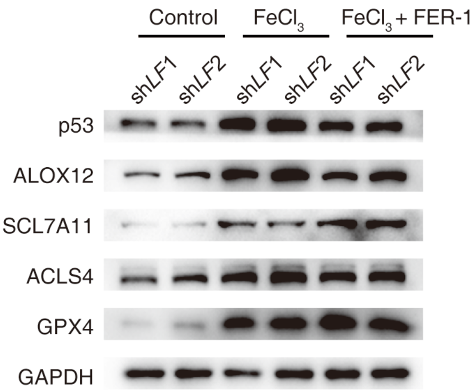

Figure S6 (related to Figure 6). The ferroptosis inhibitor FER-1 (1 μM) alleviated ferroptosis stress in *LF* knockdown (sh*LF*1/sh*LF*2) LNCaP cells. Protein expressions of p53, ALOX12, SCL7A11, ACLS4, and GPX4 were assayed by means of Western blotting.

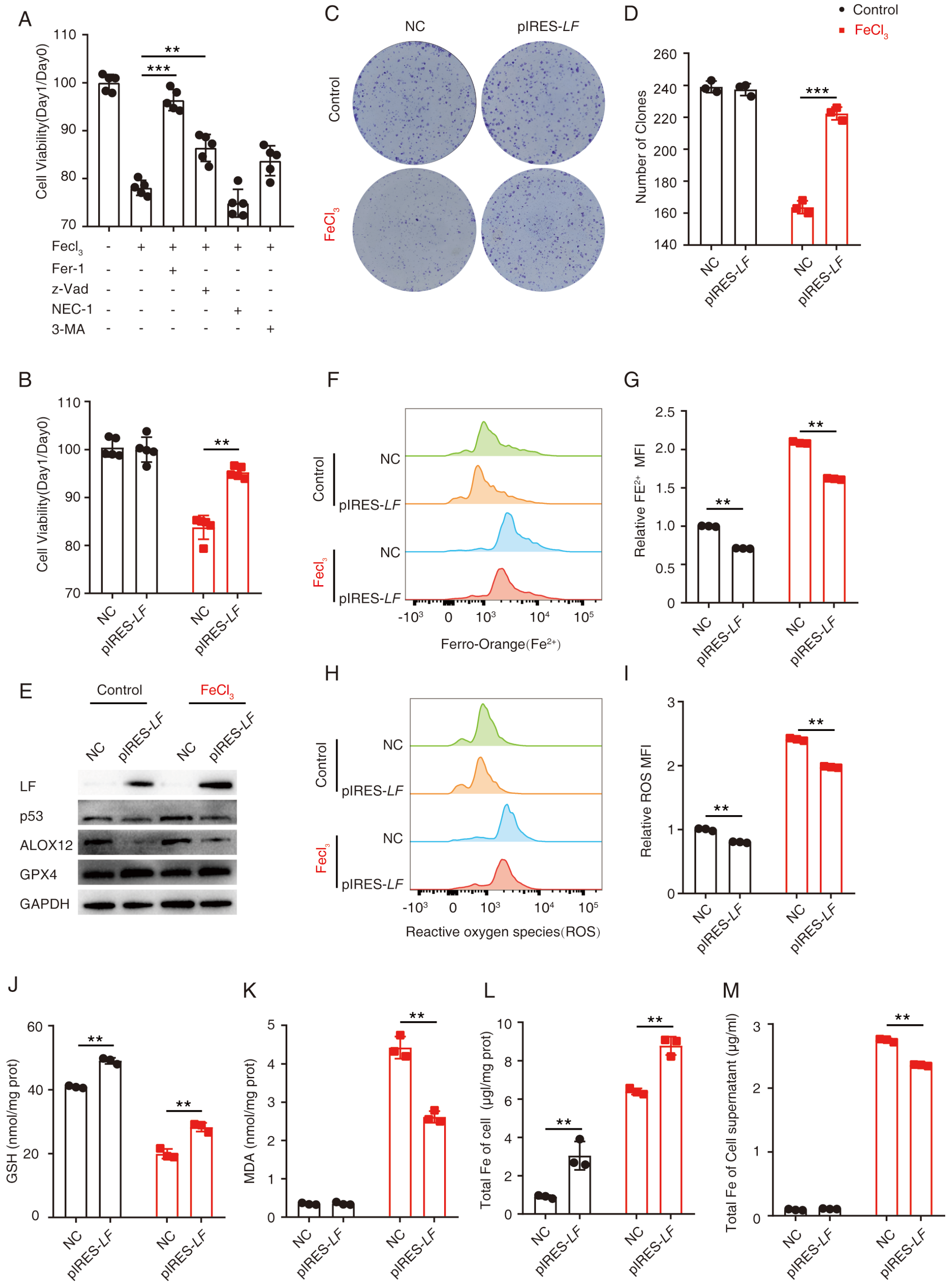

Figure S7 (related to Figure 6). *LF* overexpression attenuates ferroptosis in AR- DU145 prostate cancer cells. (A) Viability of DU145 cells treated with 100 μM $FeCl_3$ and different cell death inhibitors: Fer-1 (ferroptosis inhibitor, 1 μM), z-Vad (apoptosis inhibitor, 1 μM), Nec-1 (necroptosis inhibitor, 1 μM), 3-MA (autophagy inhibitor, 1 μM), $n = 5$. (B) Cell viability after *LF* overexpression via plasmid vectors under 100 μM $FeCl_3$, $n = 5$. (C) Colony formation capacity of *LF* overexpression DU145 cells under iron stress (100 μM $FeCl_3$), control: saline, NC: empty vectors. (D) Quantification of colony numbers, $n = 3$. (E) Protein expression of LF, p53, ALOX12, and GPX4 under iron stress. (F) Intracellular Fe^{2+} levels (FerroOrange staining) under iron stress.

(G) Quantification of Fe^{2+} fluorescence intensity, *n* = 3. (H) Intracellular ROS levels (DHE probe) under iron stress. (I) Quantification of ROS fluorescence intensity, *n* = 3. (J) Glutathione (GSH) concentration under iron stress, *n* = 3. (K) Malondialdehyde (MDA) concentration under iron stress, *n* = 3. (L) Total intracellular iron concentration under iron stress, *n* = 3. (M) Total iron content in cell culture supernatant under iron stress, *n* = 3. $p < 0.01$ "**", $p < 0.001$ "***"(one-way ANOVA for multi-group comparisons).

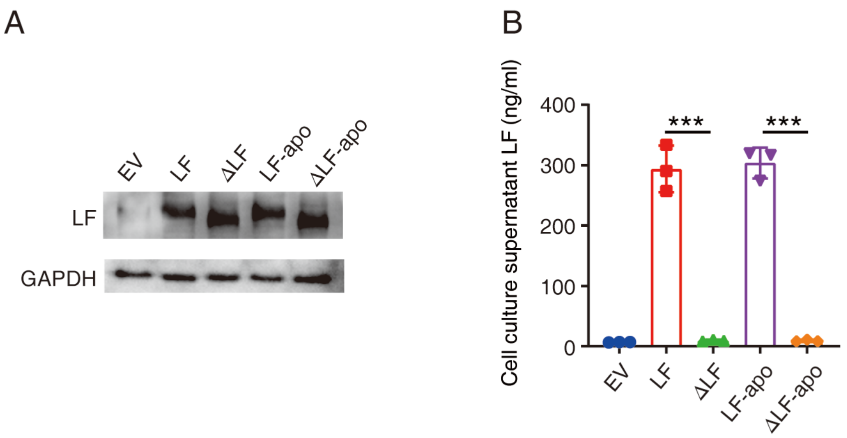

Figure S8 (related to Figure 7). Molecular validation of LF mutants. (A) Lactoferrin expression after wild-type Lactoferrin (LF), signal peptide deletion mutant (ΔLF), iron-binding domain mutant (LF-apo), or a double mutant combining both alterations(ΔLF-apo) was transfected into LNCaP-sh*LF*1 cells. (B) Lactoferrin concentration after LF, ΔLF, LF-apo, and ΔLF-apo vectors were transfected into LNCaP-sh*LF*1 cells, assayed by ELSIA. EV: empty vehicle. $p < 0.001$ "***"(one-way ANOVA for multi-group comparisons).

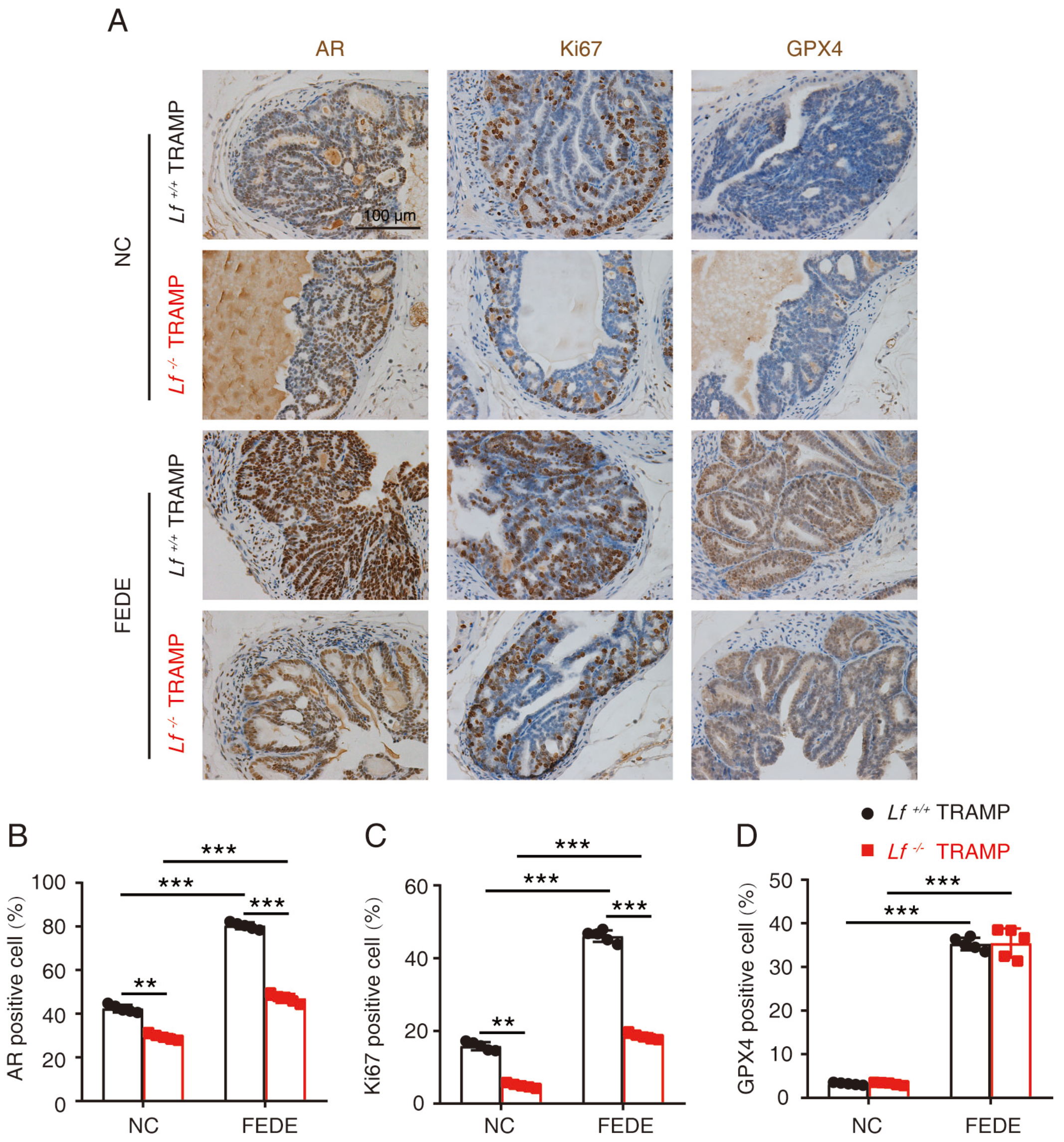

Figure S9 (related to Figure 8). AR, Ki67, and GPX4 expression profiles in iron supplementation experiments. Iron supplementation experimental design: (i) NC/TRAMP (ddH_2O i.p. in *Lf* $^{+/+}$ TRAMP mice), (ii) NC/KO (ddH_2O i.p. in *Lf* $^{-/-}$ TRAMP mice), (iii) FEDE/TRAMP (iron dextran 50 mg/kg i.p. in *Lf* $^{+/+}$ TRAMP mice), and (iv) FEDE/KO (iron dextran 50 mg/kg i.p. in *Lf* $^{-/-}$ TRAMP mice). (A) Immunohistochemistry assay of AR, Ki67, and GPX4. (B-D) Percentage quantification of AR^+, $Ki67^+$, and $GPX4^+$ cells (%) in mouse prostate tissues. $n = 5$; $p < 0.01$ "**", $p < 0.001$ "***"(one-way ANOVA for multi-group comparisons).

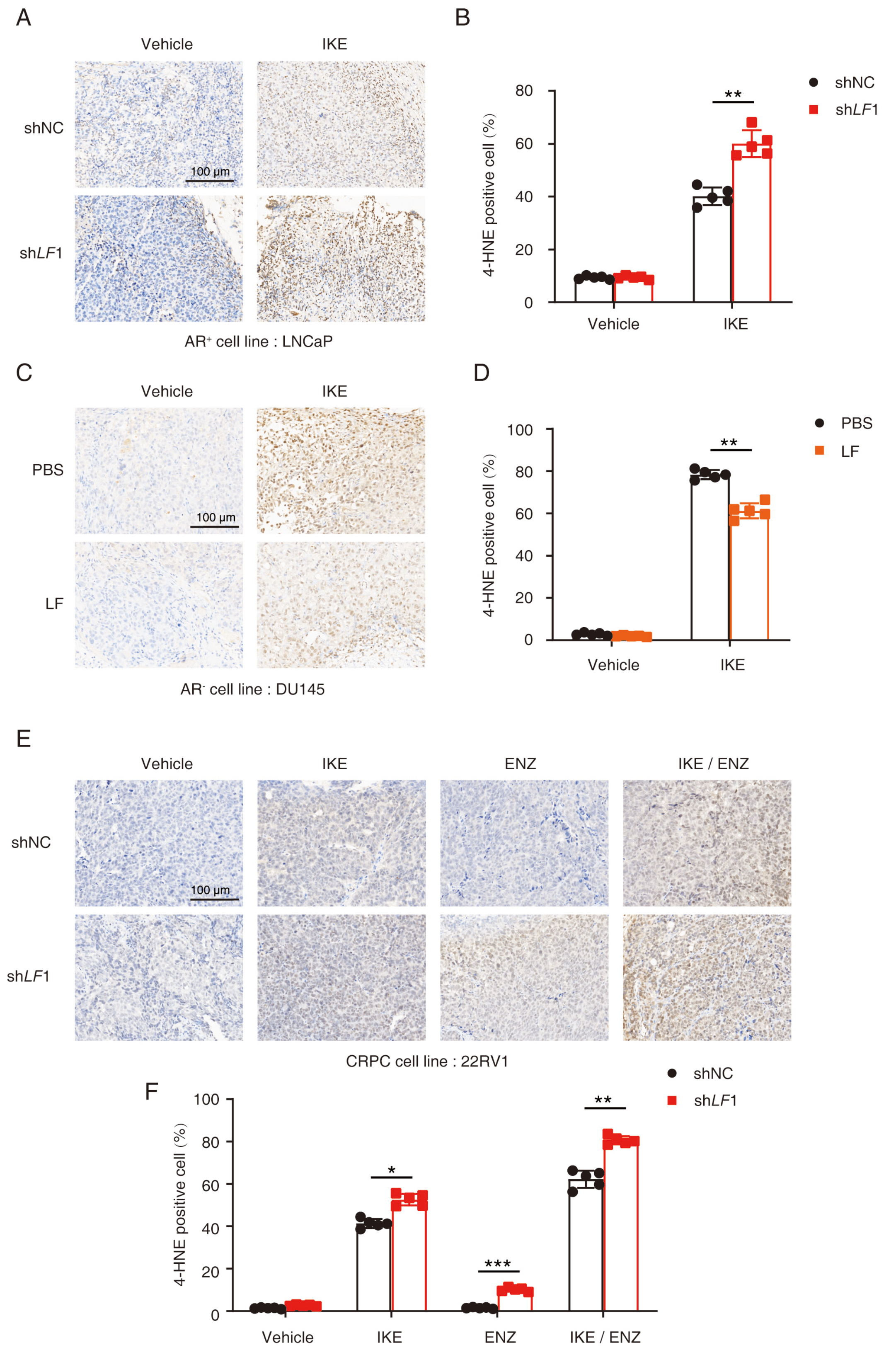

Figure S10 (related to Figure 9). Ferroptotic stress in prostate cancer xenograft models. (A) IHC staining of 4-HNE (lipid peroxidation marker) in AR^+ LNCaP xenograft model. (B) Percentage quantification of 4-HNE positive cells (%) in prostate tissue. (C) IHC staining of 4-HNE in AR^- DU145 xenograft model. (D) Percentage quantification of 4-HNE positive cells (%) in prostate tissue. (E) IHC staining of 4-HNE in CRPC 22RV1 xenograft model. (F) Percentage quantification of 4-HNE positive cells (%) in prostate tissue. *n* = 5; $p < 0.05$ "*", $p < 0.01$ "**", $p < 0.001$ "***" (one-way ANOVA for multi-group comparisons).

Supplemental Table 1. Primary antibodies used for Western Blot

**Antibody target**	**Host**	**Source**	**Catalogue #**	**Dilution**
p53	Mouse	CST	2524T	1:1000
AR	Rabbit	CST	5153S	1:1000
ACLS4	Rabbit	HUABIO	ET7111-43	1:500
DMT1	Rabbit	HUABIO	ER1907-55-50	1:500
FPN	Mouse	HUABIO	HA601178	1:500
FTH1	Rabbit	HUABIO	ET1610-78	1:500
FTL	Rabbit	HUABIO	ET1705-54	1:500
GPX4	Rabbit	HUABIO	ET1706-45	1:500
RBMS1	Rabbit	HUABIO	ER62489	1:500
SCL7A11	Rabbit	HUABIO	HA721868	1:500
STEAP3	Rabbit	HUABIO	ER64586-50	1:500
TFR1	Rabbit	HUABIO	ET1702-06	1:500
LF	Rabbit	Invitrogen	PA5-95513	1:500
GAPDH	Mouse	Proteintech	60004- I- Ig	1:2000
ALOX12	Rabbit	SAB	28678	1:500
CP	Mouse	Santa cruz	Sc-69767	

Supplemental Table 2. Primary antibodies used for Immunohistochemistry and Immunofluorescence

Antibody target	Host	Source	Catalogue #	Dilution
4-HNE	Rabbit	Abcam	ab46545	1:300
p53	Mouse	CST	2524T	1:300
AR	Rabbit	CST	HA721156	1:300
CK8	Mouse	HUABIO	M1603-2	1:300
Ki67	Rabbit	HUABIO	HA721115	1:1000
GPX4	Rabbit	HUABIO	ET1706-45	1:300
LF	Rabbit	Invitrogen	PA5-95513	1:200
SNAIL	Rabbit	Proteintech	12129-1-AP	1:500
ALOX12	Rabbit	SAB	28678	1:500
SMA	Mouse	Santa cruz	Sc-53142	1:500

Supplemental Table 3. Primers used for ChIP-PCR/qPCR

**Primers for targeted sequencing**	**Forward (5'-3)'**	**Reverse (5'-3)'**
LF Promoter 1	AGATGCTTCAGCACTCCTGG	CCTGCCATGAGACTGGGGT
LF Promoter 2	ACCCAGCTGGTGATTCTAGCAC	AGGCAATGCCCCAGGGAG
LF Promoter 3	TAGTCTAAGCAGAGTCAAGACCAGCTT	TTGAGCCTGGGAGATGAAGGTG
LF Promoter 4	CGATCCTCCCATCTTGGCCT	TTCCCATGACACACAGCACCC
LF Promoter 5	ATGGCGATTGTTAAAATGAGGTTACCA	TCCCAGAAAACAGACAGGGACCT
LF Promoter 6	ATGGAACCAGCCTTGGGTGG	CTGTGAGACTGCTATCTTCTTCAGCAG
LF Promoter 7	CTAACTGGCTCCTAGGCACCTTC	TGCACCTCACCTGTCCTGG
LF Promoter 8	AGTGGGAGAGAAAGAACATCGCAG	TGTTGCCCAATAGACACCCCT
KLK3 Promoter	CCTAGATGAAGTCTCCATGAGCTACA	GGGAGGGAGAGCTAGCACTTG
